# A sponge homolog of BRMS1 reveals ancient origin of metastasis-suppressing functions

**DOI:** 10.1186/s12915-026-02616-5

**Published:** 2026-05-05

**Authors:** Antea Talajić, Kristina Dominko, Bastien Proust, Nikolina Škrobot Vidaček, Matija Harcet, Helena Ćetković

**Affiliations:** 1 Division of Molecular Biology, Laboratory for Molecular Genetics, Ruđer Bošković Institute, Zagreb, 10000 Croatia; 2 Division for Toxicology, Croatian Institute of Public Health, Zagreb, 10000 Croatia

**Keywords:** Porifera, Early metazoans, BRMS1, Evolution, Metastasis suppressor, Cell migration, Intracellular localization, Cancer biology

## Abstract

**Background:**

Early-branching metazoans, such as sponges (Porifera), can provide valuable insight into the emergence of complex gene regulatory systems and cancer-related mechanisms in early metazoan evolution. BRMS1 (breast cancer metastasis suppressor 1) is a component of the Sin3-HDAC complex involved in chromatin remodeling and transcriptional regulation. In humans, BRMS1 inhibits cancer metastasis by modulating signaling pathways that control cell migration, adhesion, and proliferation. Despite its biomedical importance, the evolutionary origin and basic functions of BRMS1 remain largely unexplored.

**Results:**

We identified and characterized a BRMS1 homolog from the cave sponge *Eunapius subterraneus* and compared it with human BRMS1 and BRMS1-like paralogs. Phylogenetic analyses revealed that BRMS1 and BRMS1-like arose from a duplication of an ancestral BRMS1 gene during early vertebrate evolution. Structural modeling showed that sponge BRMS1 shares high similarity with human BRMS1. Co-immunoprecipitation assays demonstrated that sponge BRMS1 physically associates with human BRMS1 in mammalian cells. In both sponge and human cells, sponge BRMS1 localized predominantly to the nucleus, similar to human BRMS1 and BRMS1-like. Functional assays in human breast cancer cells revealed that sponge BRMS1 suppresses proliferation, colony formation, and migration to a degree comparable to its human homologs.

**Conclusions:**

Our findings demonstrate that the key structural features, subcellular localization, and biological functions of BRMS1 are conserved between sponges and humans. The ability of a sponge BRMS1 homolog to integrate into human protein complexes and suppress cancer cell migration and proliferation suggests that fundamental BRMS1 activities arose early in metazoan evolution, independent of anatomical and functional complexity.

**Supplementary Information:**

The online version contains supplementary material available at 10.1186/s12915-026-02616-5.

## Background

Sponges (Porifera) are an excellent model for studying the origin and early evolution of Metazoa, as they are an early-branching metazoan lineage [1] with relatively few cell types and simple morphology. In contrast to that, sponges possess complex genomes [2–4] and contain most of the genes present in highly complex animals such as vertebrates, including genes involved in signaling pathways, early development, and carcinogenesis [2, 5]. Although they possess many gene homologs associated with tumor initiation and progression in humans [6–17], tumors have not been identified in sponges. This might be due to their simple anatomy being incompatible with tumorigenesis, resistance to tumorigenic agents [18], or to our knowledge gap, i.e., the lack of systematic studies and understanding of sponge diseases. Given that many sponge genes are highly similar to their vertebrate homologs [19], comparative studies that include sponges offer valuable insights into the genomic and proteomic characteristics of the common ancestor of all Metazoa [2, 11, 19]. It has been shown previously that at least some sponge homologs, such as NME1, FAU, DRG1, and RRAS2, exhibit biochemical and biological properties akin to those of human proteins involved in cancer [14, 15, 20, 21]. These studies suggest that the properties associated with cancer development and progression in the aforementioned proteins may be linked to fundamental cellular functions, which seem to be important for both simple metazoans and humans.

The BRMS1 (breast cancer metastasis suppressor 1) protein is a component of the Sin3-HDAC complex, which is involved in chromatin remodeling and the regulation of gene transcription [22]. Sin3-HDAC complexes are evolutionarily conserved between budding yeast, *Saccharomyces cerevisiae*, and human (*Homo sapiens)*, and have a function in numerous cellular processes, including cell proliferation, differentiation, aging, and apoptosis [23, 24]. These complexes also represent important therapeutic targets for the treatment of several cancers, including triple-negative breast cancer and pancreatic cancer [25, 26]. BRMS1 shares an evolutionarily conserved Sds3-like domain with the SDS3 and BRMS1-like proteins, which is why these three proteins are often considered members of the same protein family [27–30]. All three proteins of the BRMS1 family are members of the Sin3-HDAC complex and share some structural similarities. The BRMS1 shares 49% sequence similarity with the SDS3 and 79% with the BRMS1-like [30, 31]. While BRMS1 and BRMS1-like suppress metastasis in several types of cancer, SDS3 lacks metastasis-suppressing activity [22, 30, 32, 33]. Notably, BRMS1-like has been shown to inhibit epithelial-mesenchymal transition (EMT) and may allow functional compensation when BRMS1 expression is epigenetically silenced [28, 31]. Unlike BRMS1, BRMS1-like and SDS3 harbor a C-terminal capped Tudor domain (CTD), highlighting structural diversification within this protein family [32].

In humans, BRMS1 is encoded on chromosome 11q13, a region frequently deleted in cancers, including breast cancer and melanoma. The loss of BRMS1 is correlated with poor survival [33, 34]. Human BRMS1 comprises 246 amino acids and consists of a glutamate-rich region, two coiled-coil domains, two nuclear localization signals (NLS), a succession of partial/atypical leucine zipper motifs, and a nuclear export signal (NES) [33, 35]. Its expression is regulated at multiple levels: transcriptionally, post-transcriptionally, post-translationally, and via degradation [36–38]. BRMS1 influences several signaling pathways, including EGFR, NF-κB, and possibly FAK [33].

The key activity of BRMS1 involves the inhibition of the NF-κB signaling pathway by reducing the expression of metastatic proteins such as uPA, CXCR4, and OPN, which influence cell migration and invasion [39–41]. In addition, BRMS1 negatively regulates the EGFR/PI3K/Akt signaling pathway, resulting in reduced cell motility and metastatic ability [42, 43]. It has been proposed that the BRMS1 protein also affects the FAK/Src signaling pathway, which is important for the regulation of focal adhesions, although the mechanism is not yet fully understood [33].

Initially identified as a metastasis suppressor in breast cancer (MDA-MB-231) and melanoma (MDA-MB-435) cells [34], BRMS1 has since been shown to inhibit metastasis in diverse cancers without significantly affecting primary tumor growth [33, 44, 45]. It acts at multiple steps of the metastatic cascade, including invasion, migration, adhesion, and colonization, most often through NF-κB–dependent mechanisms [33, 44].

In contrast to increasing functional insights, the evolutionary history of the BRMS1 protein family remains largely unexplored, limiting our understanding of how structural features relate to molecular activity and biological roles across species. Previous comparative genomic studies revealed that members of this family are widely distributed across Metazoa and their close unicellular relatives, with notable absences in *Monosiga brevicollis* (choanoflagellate), *Caenorhabditis elegans* (nematode), and *Crassostrea gigas* (Mollusca) [11]. However, beyond such surveys, the phylogenetic relationships among BRMS1, BRMS1-like, and SDS3 remain poorly resolved, and little is known about how their functions originated and diversified during early metazoan evolution. This knowledge gap is particularly striking given the important role of the BRMS1 protein in cancer biology and metastasis suppression in humans.

Early-branching metazoan lineages, such as sponges (Porifera), represent valuable models for addressing these questions. Sponges occupy a key phylogenetic position for understanding metazoan evolution, have a wide gene repertoire, and share numerous homologs with humans, including components of pathways related to cell signaling, adhesion, and tumor-associated processes [2, 4, 12, 19]. Studying BRMS1 homologs in sponges may therefore provide important insights into the basic and conserved functions of this protein family, and contribute to understanding the evolutionary origins of its metastasis-suppressing properties.

The aim of this study was to elucidate the evolutionary history of the BRMS1 protein family, with a particular focus on the metastasis suppressor BRMS1 in Metazoa. We examined the evolutionary relationships among BRMS1, BRMS1-like, and SDS3 across different metazoan phyla. Further, we analyzed the biological roles of BRMS1 protein through the characterization of human and sponge homologs, thereby providing novel insights into the conserved functions and biological roles of this important protein family.

## Results

### Evolutionary history of the BRMS1 protein

**Fig. 1:**
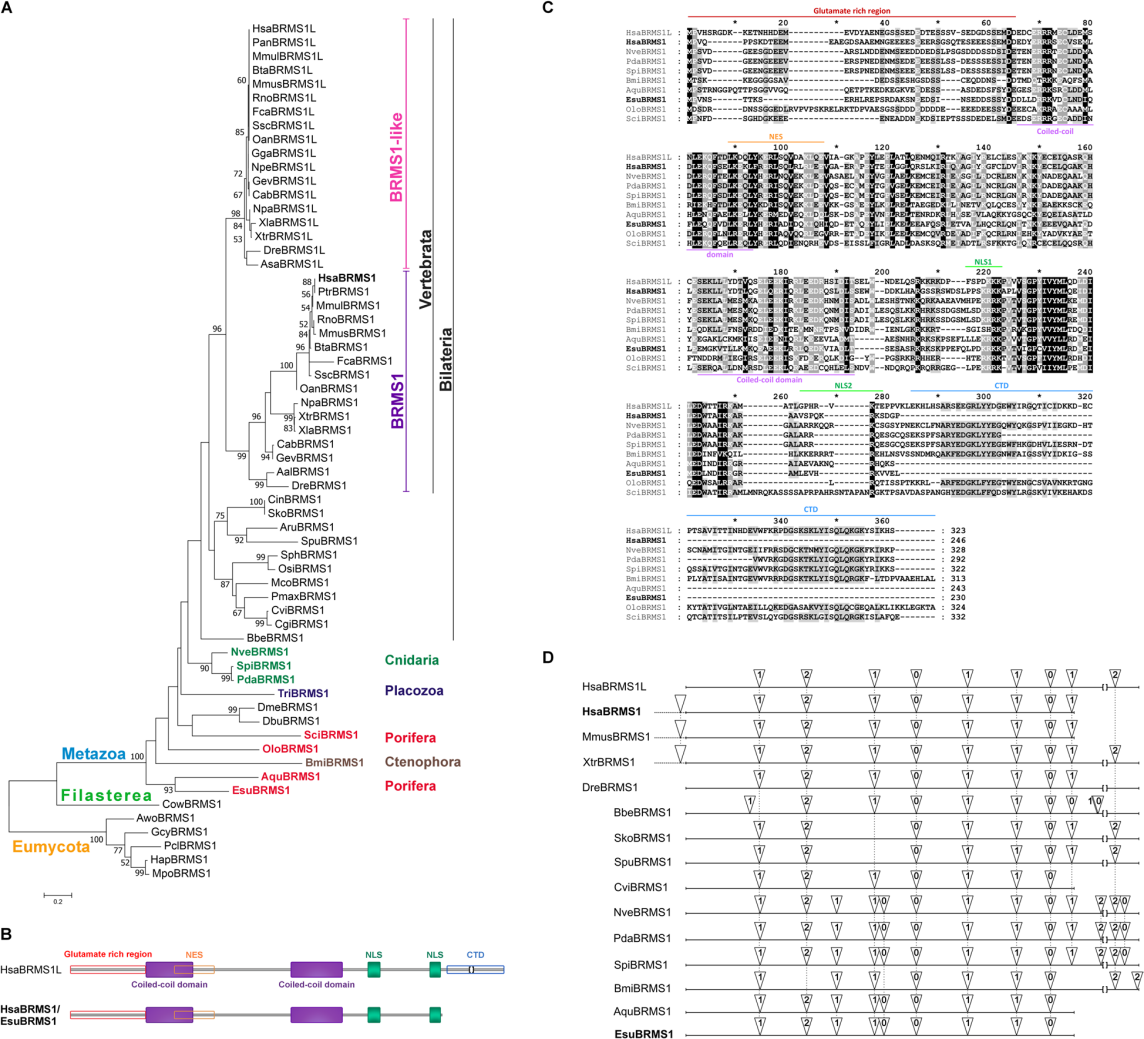
Evolutionary analysis of the BRMS1 protein family. **A** Phylogenetic analysis of BRMS1 and BRMS1-like proteins from Metazoa, Filasterea, and Eumycota. The phylogenetic tree was constructed using the maximum likelihood method (JTT + G model) and includes 62 amino acid sequences. Bootstrap support values, based on 1000 replicates, are shown at the branching points (only values > 50% are displayed). The scale bar indicates the number of substitutions per site. Representatives of Eumycota, Filasterea, and Metazoa are shown in orange, green, and blue, respectively. Within Metazoa, vertebrate BRMS1 proteins are highlighted in purple and vertebrate BRMS1-like proteins in pink. Non-bilaterian metazoan BRMS1 proteins are color-coded as follows: Cnidaria (dark green), Placozoa (dark blue), Ctenophora (brown), and Porifera (red). **B** Schematic representation of human and sponge BRMS1 and BRMS1-like proteins with conserved domains indicated. **C** Multiple sequence alignment of BRMS1 and BRMS1-like proteins from Porifera, Ctenophora, Cnidaria, and humans. Conserved domains are marked as follows: glutamate-rich region (red, above the alignment), coiled-coil domains (purple, below the alignment), nuclear export signal (NES; orange, above the alignment), nuclear localization signals NLS1 and NLS2 (green, above the alignment), and the C-terminal capped Tudor domain (CTD; blue, above the alignment). **D** Gene structures of *brms1* and *brms1*-like in selected metazoans. Triangles mark intron positions, with numbers indicating intron phases. Black dashed lines connect introns that share the same position and phase, based on the alignment of amino acid sequences. Gene models with intron positions were obtained from the NCBI genomic database. Species abbreviations, amino acid sequences, and accession numbers are listed in Additional file 2

To elucidate the evolutionary history of the BRMS1 protein, we performed a maximum likelihood phylogenetic analysis (JTT + G model) including representatives from Eumycota, Filasterea, and Metazoa. The tree is rooted using Eumycota BRMS1 sequences as an outgroup. BRMS1 representatives from Eumycota, Filasterea, and Metazoa clustered into distinct, well-supported clades (bootstrap support of 100%). The analysis revealed a gene duplication event that likely occurred at the origin of vertebrates (bootstrap support 96%), giving rise to two distinct paralogs, BRMS1 and BRMS1-like (Fig. 1A). Both vertebrate paralog clades are strongly supported (bootstrap support 99–100%). In contrast, Filasterea and non-vertebrate metazoans possess a single BRMS1 protein, indicating that the ancestral genome likely possessed a single *brms1* gene prior to vertebrate diversification. Although major taxonomic groups are generally well resolved, deep relationships among non-bilaterian metazoans (Porifera, Ctenophora, Cnidaria, and Placozoa) remain weakly supported (bootstrap support < 50%). The placement of certain bilaterian taxa (*Drosophila* spp.) as sister to non-bilaterian clades may reflect relatively rapid molecular evolution characteristic for this dipteran lineage, which can reduce the reliability of deep phylogenetic inference [46]. Consequently, the present dataset does not allow reliable inference regarding whether Porifera or Ctenophora represents the earliest-diverging metazoan lineage.

To gain further insight into the evolutionary history of the BRMS1 protein family, we included the SDS3 protein into our extended phylogenetic analysis. In the resulting tree (Additional file 1: Fig. S1), Eumycota SDS3 and BRMS1 cluster as sister branches within a clade clearly separate from metazoan proteins. Within Metazoa, SDS3 and BRMS1 also form distinct, well-supported branches (bootstrap support of 70% and 89%, respectively), indicating an early duplication event giving rise to these paralogs. The topology is consistent with SDS3 and BRMS1 representing ancient paralogs already present in the common ancestor of Metazoa, with independent lineage-specific divergence in Eumycota and Metazoa. BRMS1-like is restricted to vertebrates and likely originated from a duplication of BRMS1, reflecting a vertebrate-specific paralogous expansion within this protein family. While the precise relationship between fungal and metazoan BRMS1 homologs cannot be fully resolved in this analysis, the tree suggests deep divergence between Eumycota and Metazoa homologs.

To examine the conservation of protein sequences, we compared BRMS1 and BRMS1-like proteins from sponges, ctenophores, cnidarians, and humans. A schematic representation of these proteins, showing their characteristic domains, is presented in Fig. 1B. The multiple sequence alignment (Fig. 1C) shows key BRMS1 domains: a glutamate-rich region, two coiled-coil domains, a nuclear export signal (NES), and two nuclear localization signals (NLS1 and NLS2). Human BRMS1-like additionally contains a C-terminal capped Tudor domain (CTD). This domain is absent from human BRMS1 and BRMS1 homologs of demosponges (class Demospongiae, Porifera). BRMS1 from other sponge classes (Calcarea and Homoscleromorpha, Porifera), Ctenophora, and Cnidaria possess a partially conserved CTD. Although BRMS1 sequences are not highly conserved between sponges, ctenophores, cnidarians, and humans, several structural domains, including the coiled-coil domains and the NES and NLS1 motifs, are retained across these lineages (see Fig. 1C), suggesting functional relevance in chromatin regulation and protein–protein interactions. The conservation of these domains between human BRMS1 and BRMS1-like paralogs further supports their shared participation in the same chromatin remodeling complex, likely requiring common binding partners and overlapping cellular functions.

To further analyze their similarity, we compared the complete sequences of BRMS1 and BRMS1-like proteins across metazoans and Filasterea, visualized using a heatmap (Additional file 1: Fig. S2). Sequence similarity is highest among vertebrate BRMS1-like proteins (72–100%), reflecting their recent origin, while vertebrate BRMS1 proteins show somewhat lower conservation (50.7–98.8%) (Additional file 3). Human BRMS1 and BRMS1-like share 55% similarity. Filasterea and invertebrates possess a single BRMS1 protein, showing comparable similarity to both vertebrate paralogs. Demosponge BRMS1 proteins are more similar to human BRMS1 (*Amphimedon queenslandica* 49%, *Eunapius subterraneus* 52%) than to human BRMS1-like (*A. queenslandica* 38%, *E. subterraneus* 41%), consistent with their comparable protein lengths and the absence of a C-terminal capped Tudor domain. Despite low overall sequence conservation across distant taxa (27–87%), key domains are maintained, highlighting their functional importance.

Analysis of intron–exon organization of *brms1* and *brms1-like* genes in selected metazoans revealed that all eight introns present in the human *brms1* gene are also found in sponges and cnidarians, with seven conserved in sponge homologs (Fig. 1D). An intron located upstream of the start codon was detected exclusively in vertebrate *brms1* genes. Both human paralogs, *brms1* and *brms1-like*, contain nine introns, sharing the position and phase of eight, consistent with a relatively recent duplication event. Importantly, human *brms1-like* contains an additional intron at the 3′ end, absent from most vertebrate *brms1* genes, except in *Xenopus laevis*, where an extended 3′ region harbors this intron. These findings indicate that the intron-rich structure of the *brms1* gene is evolutionarily conserved and likely derives from a homolog already present in the last common ancestor of Metazoa.

### Sponge and human BRMS1 show structural similarity and coexist within the same complexes

**Fig. 2:**
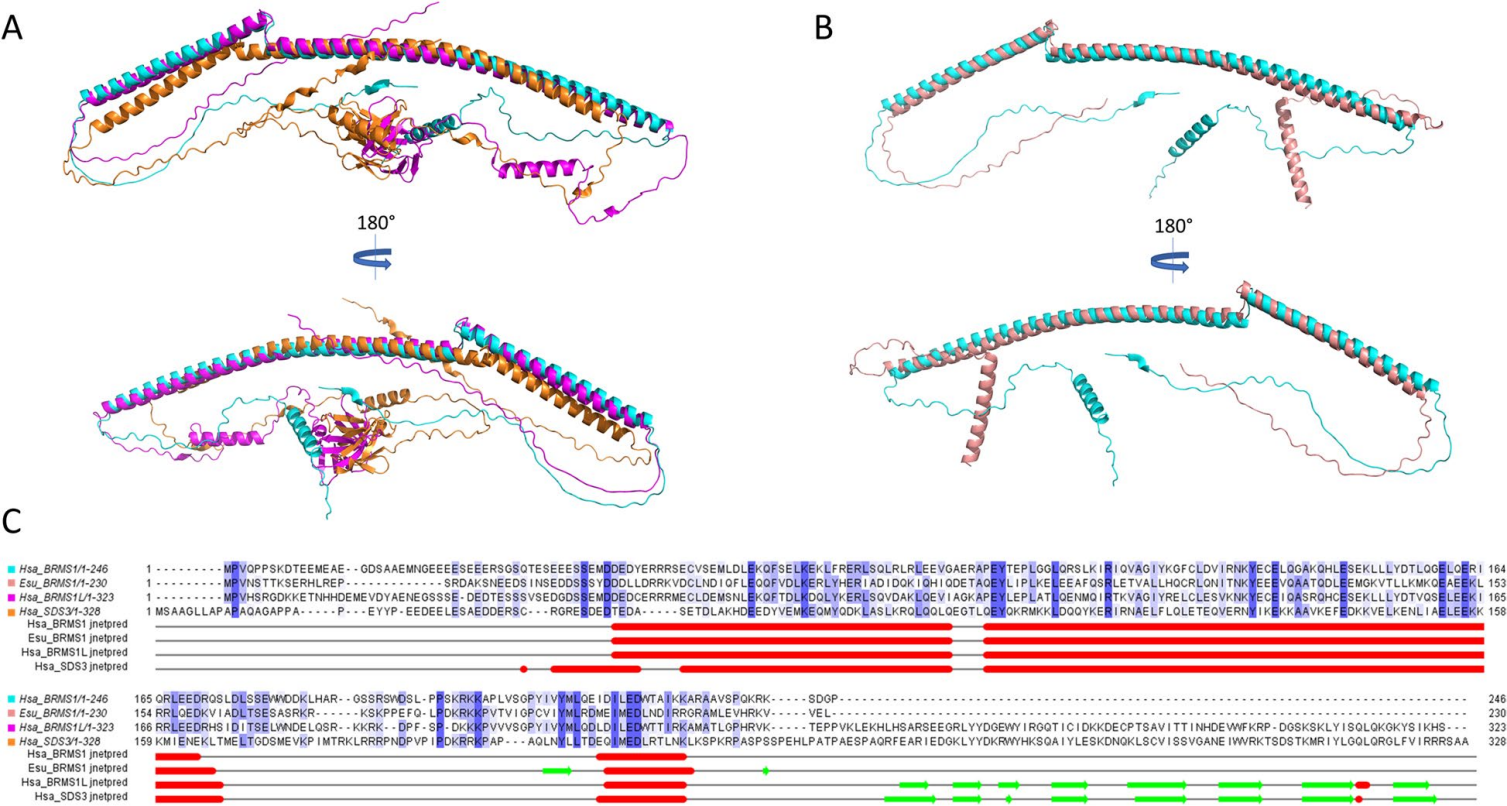
Sponge and human BRMS1 show structural similarity. **A** Superimposition of AlphaFold predicted 3D structures from HsaBRMS1 (cyan, AF-Q9HCU9-F1), HsaBRMS1-like (magenta, AF-Q5PSV4-F1), and HsaSDS3 (orange, AF-Q9H7L9-F1) performed on Pymol. **B** Superimposition of predicted 3D structures from HsaBRMS1 (cyan, AF-Q9HCU9-F1) and EsuBRMS1 (salmon, internally predicted using Alphafold3) performed on Pymol. **C** Sequence alignment of HsaBRMS1, EsuBRMS1, HsaBRMS1-like, and HsaSDS3 (Jalview, ClustalO alignment default settings), with sequences colored by sequence identity (cutoff 25%). The secondary structures were predicted for each protein independently (Jalview, secondary structure prediction, JPRED4). The red line represents the alpha-helix while the green arrow represents beta-sheets. Abbreviations: Esu, *Eunapius subterraneus*; Hsa, *Homo sapiens*

The comparison of structures predicted (Alphafold3) from human HsaBRMS1, HsaBRMS1-like, sponge EsuBRMS1, and human HsaSDS3 revealed interesting similarities between proteins (Fig. 2). These four proteins have the following architecture: starting from the N-terminus, a disordered region (40 to 50 aa) is followed by a first alpha-helix (~ 40 aa). A small loop (4–5 aa) is connected to a second alpha-helix (~ 75 aa), followed by a longer disordered region (40–50 aa) leading to a third, shorter, alpha-helix (~ 12 aa). From this point, we can separate proteins into two groups: HsaBRMS1 and EsuBRMS1 end shortly after the third alpha-helix (Fig. 2B), while HsaBRMS1-like and HsaSDS3 have an extension (~ 100 aa long) folding as a succession of antiparallel beta-sheets assembling as the C-terminal capped Tudor domain (CTD) (Fig. 2A). The four proteins are enriched in charged and polar residues, with glutamate (E) being the most abundant and lysine (K), arginine (R), and aspartate (D) as well as leucine (L) scoring in the top 6 most abundant. Noteworthy, among the 21 amino acids conserved across the four proteins (Fig. 2C), 16 are charged or polar and most of them are located within one of the three alpha-helixes. Moreover, the residues located in the first and second alpha-helixes predominantly face the same direction (or lie in the same plane), suggesting a specific conserved interaction surface (Additional file 1: Fig. S3). Computed comparison of predicted structures showed that the BRMS1 protein from the sponge is more similar to human BRMS1 than to human BRMS1-like (Additional file 1: Table S1), largely imputable to the C-terminal extension of HsaBRMS1L (Fig. 2C), which is in agreement with the results of our phylogenetic analysis.

Crystallographic analysis of the N-terminal coiled-coil region of human BRMS1 [47], along with in silico predictions (Additional file 1: Fig. S4), suggests that the protein might oligomerize as an antiparallel dimer (pTM = 0.46; ipTM = 0.46; pLDDT: high), while an hexameric organization seems significantly less plausible (pTM = 0.22; ipTM = 0.17; pLDDT: moderate/low). Structural modeling supports the idea that both EsuBRMS1 and HsaBRMS1 share conserved coiled-coil domains that may facilitate homo- or hetero-oligomerization.

**Fig. 3:**
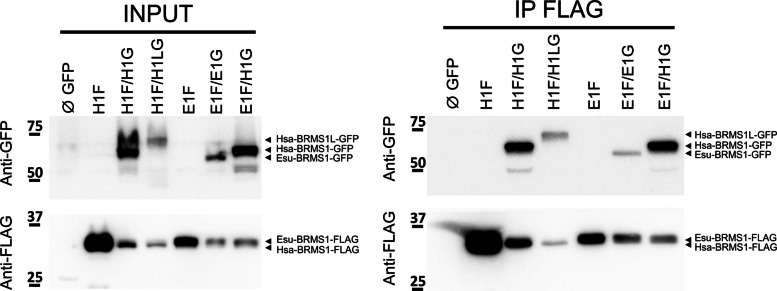
Sponge BRMS1 interacts with itself and human BRMS1. Similarly, human BRMS1 interacts with both itself and BRMS1-like. MCF7 cells were (co)transfected with plasmids encoding empty GFP (∅ GFP); human proteins HsaBRMS1-FLAG (H1F), HsaBRMS1-GFP (H1G), and HsaBRMS1-like-GFP (H1LG); or sponge proteins EsuBRMS1-FLAG (E1F) and EsuBRMS1-GFP (E1G). Co-immunoprecipitated proteins were analyzed by western blot using anti-FLAG and anti-GFP antibodies. Abbreviations: Esu, *Eunapius subterraneus*; Hsa, *Homo sapiens*

So far, native HsaBRMS1 and EsuBRMS1 proteins have not been successfully isolated and purified in quantities sufficient for biochemical characterization. To investigate potential interactions of EsuBRMS1, HsaBRMS1, and HsaBRMS1-like proteins under physiological conditions, co-immunoprecipitation (co-IP) assays were performed in MCF7 (Fig. 3), MDA-MB-231T, and HEK293 cells (Additional file 1: Fig. S5) transiently expressing the proteins, either individually or in combination. The results demonstrated that the Flag-tagged sponge EsuBRMS1 co-immunoprecipitates both with itself and with the human BRMS1 when co-expressed in human cells. Similarly, the Flag-tagged human BRMS1 could pull-down both itself and BRMS1-like, confirming that these proteins coexist within the same complexes. Together, these findings indicate that EsuBRMS1 can interact with its human homolog and participate in the same protein assemblies within mammalian cells.

### The sponge BRMS1 and human BRMS1 and BRMS1-like have identical localization

**Fig. 4:**
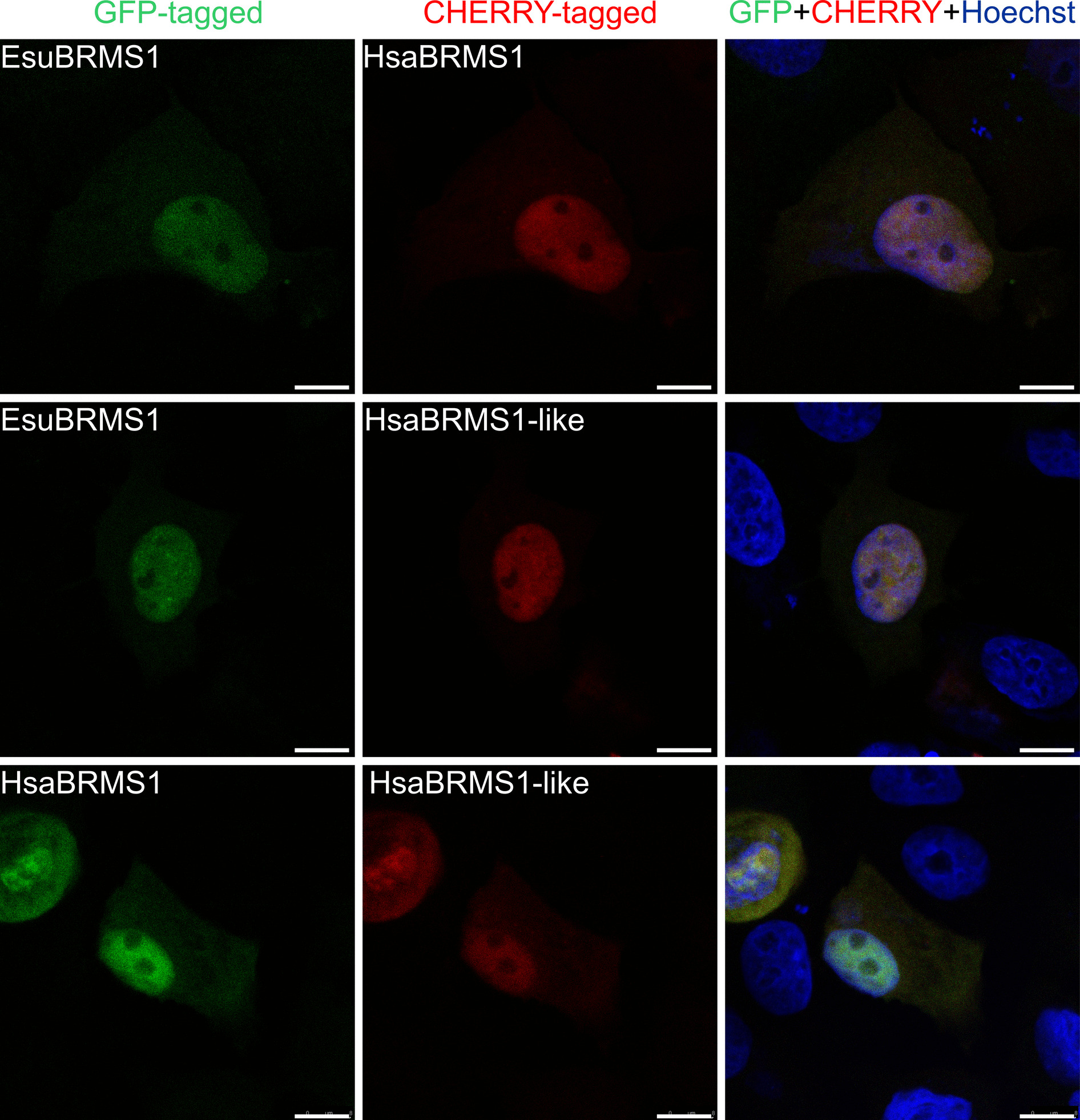
The subcellular localization of the proteins EsuBRMS1, HsaBRMS1, and HsaBRMS1-like is predominantly nuclear, but also present in the cytoplasm of human MCF-7 tumor cells. In the upper panel, EsuBRMS1 was labeled with GFP (green), and HsaBRMS1 with mCherry (red). In the middle panel, EsuBRMS1 was labeled with GFP (green), and HsaBRMS1-like with mCherry (red). In the lower panel, HsaBRMS1 was labeled with GFP (green), and HsaBRMS1-like with mCherry (red). Protein colocalization is indicated by yellow fluorescence. Nuclei were counterstained with Hoechst (blue). Images were aquired by confocal microscopy. Transfection efficiency > 50%. Scale bar represents 10 µm. Abbreviations: Esu, *Eunapius subterraneus*; Hsa, *Homo sapiens*

For the detection of intracellular localization of sponge EsuBRMS1, human HsaBRMS1, and HsaBRMS1-like proteins, MCF-7 cells were transfected with constructs of interest labeled with GFP (Fig. 4, left column) or CHERRY (Fig. 4, middle column). We found that all three proteins are mainly present in the nucleus. However, a weak specific signal is also present in the cytoplasm. The co-transfection showed a complete colocalization of the EsuBRMS1 protein with the HsaBRMS1 and HsaBRMS1-like proteins, as well as colocalization of the HsaBRMS1 protein with the HsaBRMS1-like protein. The obtained results confirmed the identical localization of the sponge and human BRMS1 proteins and the human BRMS1-like protein mainly in the nucleus and partially in the cytoplasm of human MCF-7 tumor cells. Furthermore, we transfected HeLa cells with GFP-tagged EsuBRMS1 or HsaBRMS1 proteins. The results revealed that both BRMS1 homologs localize predominantly in the nucleus, with additional presence in the cytoplasm (Additional file 1: Fig. S6). This findings confirm the similar subcellular localization of these proteins in the human tumor cell lines MCF-7 and HeLa.

**Fig. 5:**
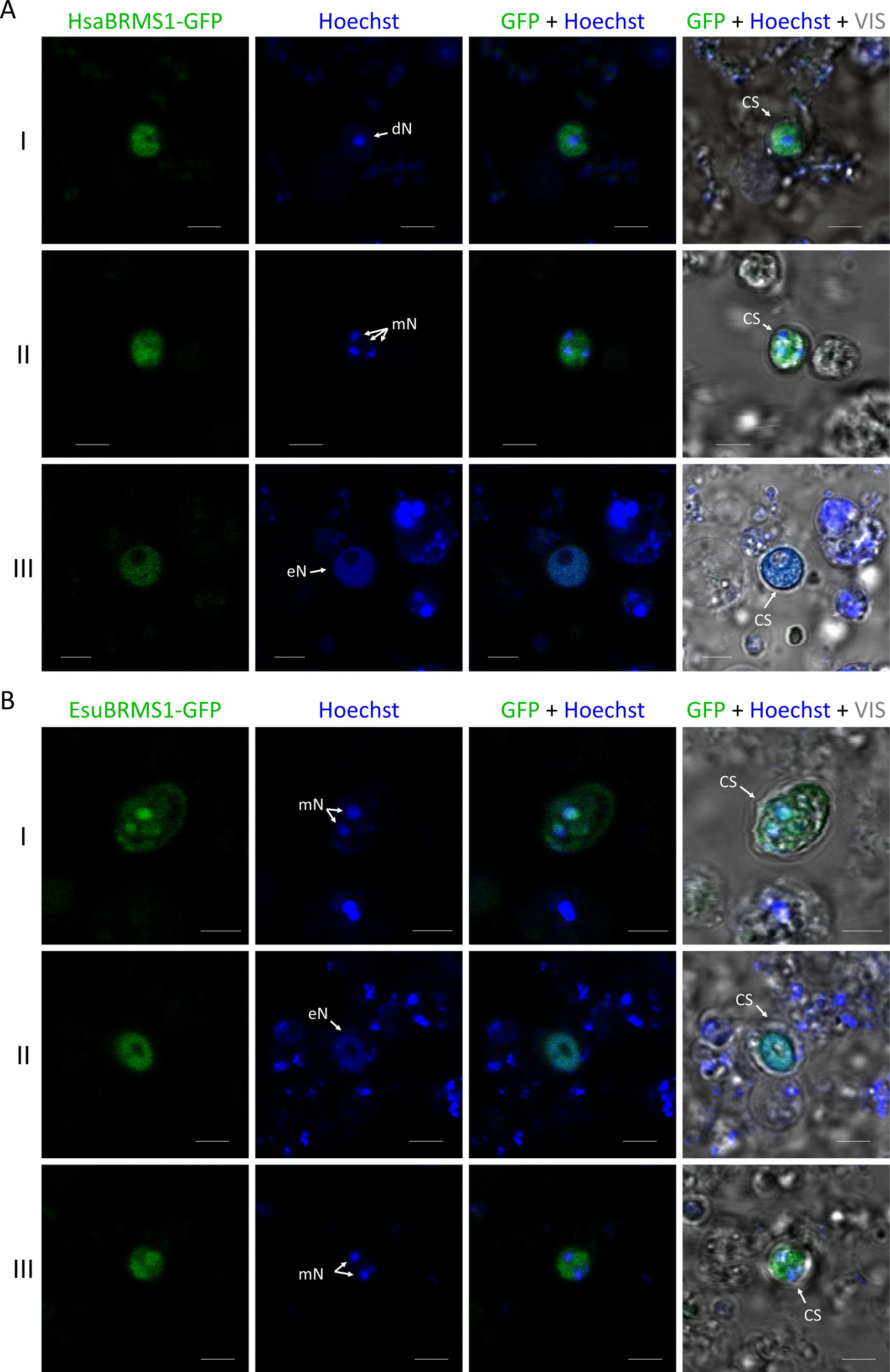
Sponge and human BRMS1 homologs were localized in the nucleus and the cytoplasm of live sponge cells. Cells were transfected with **A** HsaBRMS1 and **B** EsuBRMS1 tagged with GFP. Nuclei were counterstained with Hoechst (blue). Different shapes of nuclei can be observed: discrete nucleus (dN); multi-lobbed nucleus (mN); and extended nucleus (eN). Images were acquired by confocal microscopy. Cell surfaces (CS) were annotated based on the VIS channel. Transfection efficiency < 1%. Scale bar represents 3 µm. Abbreviations: Esu, *Eunapius subterraneus*; Hsa, *Homo sapiens*

To determine the intracellular localization of BRMS1 homologs from sponges and humans in cells of the sponge *E. subterraneus*, sponge cells were transfected with constructs for EsuBRMS1 or HsaBRMS1 proteins labeled with the fluorescent marker GFP. Since there are no optimized protocols for sponge cell culture and transfection, we came across a number of methodological and technical difficulties that could have an effect on the quality of images and precise determination of protein localization, including transfection of sponge cells in suspension and acquiring live-cell confocal images without fixing the cells first. Despite the low transfection efficiency (< 1%), a known feature of sponge cell transfection (Additional file 1: Fig. S7), cells with a visible GFP signal were successfully identified, confirming the expression of the BRMS1 protein in some of the sponge cells. The results show that the HsaBRMS1 (Fig. 5A) and EsuBRMS1 (Fig. 5B) proteins partially colocalize with Hoechst, suggesting that both proteins are localized in the nucleus and the cytoplasm of sponge cells. Although these experiments need further clarification, they provide important additional information, consistent with the previously determined localization of the BRMS1 protein from sponge and human in the human tumor cell lines MCF-7 and HeLa.

### Main cancer-related functions of the sponge and human BRMS1 are conserved

**Fig. 6:**
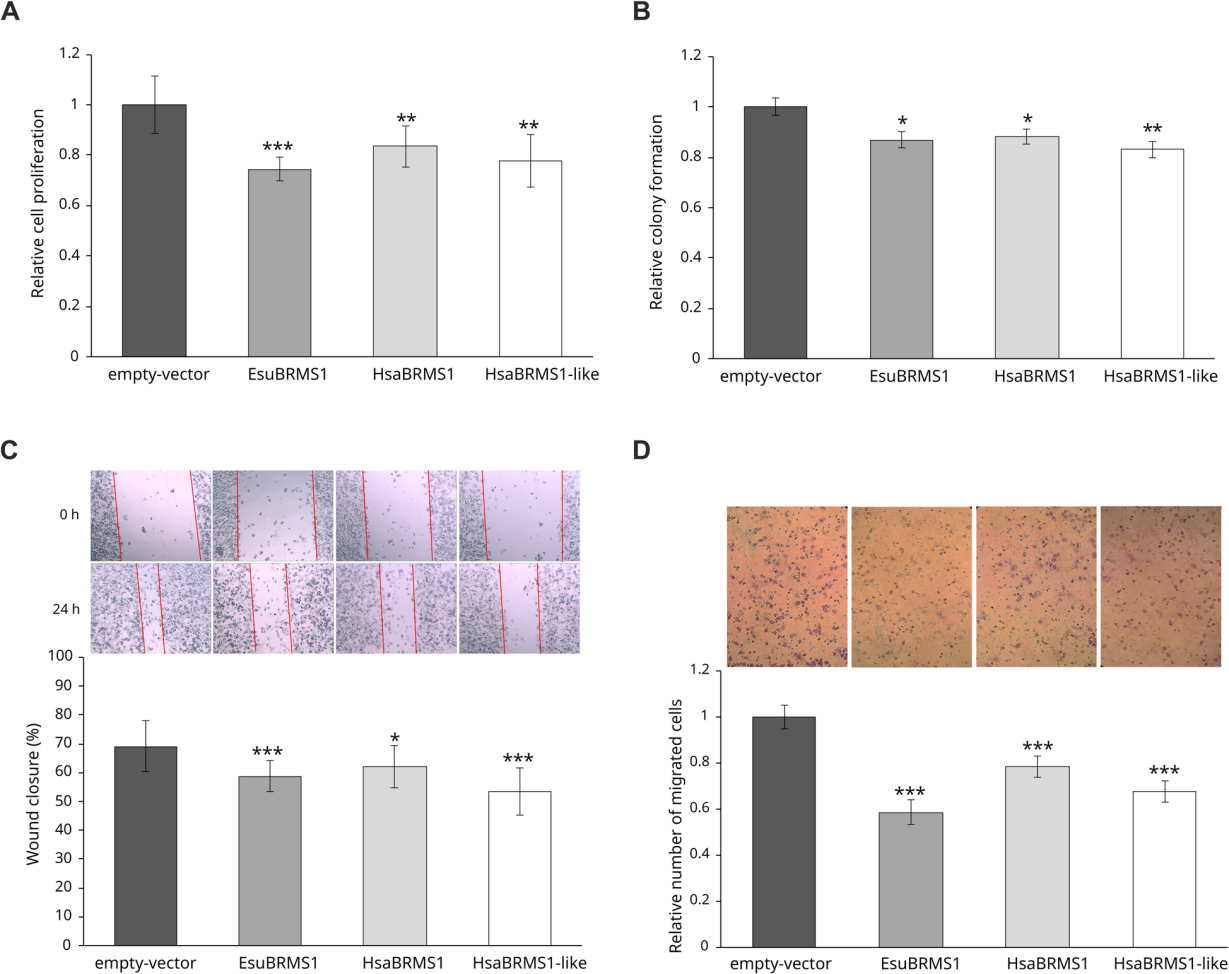
Effect of EsuBRMS1, HsaBRMS1, and HsaBRMS1-like overexpression on fundamental biological processes related to cancer in MDA-MB-231 cells. **A** Cell proliferation was assessed by MTT assay and normalized upon the absorbance value of the control (empty vector). **B** Colony formation was evaluated by counting Giemsa-stained colonies after 10 days of G418 selection and normalized to the empty vector control. **C** Wound healing assay: representative wound gap width upon cell-monolayer scratching (T = 0 h) and after 24 h (magnification 100 ×). Red lines indicate the edges of the migration front. The wound closure represents the cell-free area remaining after 24 h (0%: T24h_area = T0h_area; 100%: T24h_area = null). All areas were quantified using ImageJ software (NIH). **D** Cell migration assay: Boyden chamber membranes were captured at 200 × magnification after 24 h of migration toward FBS. Migrated cells were quantified using ImageJ (NIH), normalized upon control (empty vector), and presented as bar graphs. Each result was compared with control cells (pcDNA3) using Student’s *t*-test. Statistical significance was defined as *p < 0.05, **p < 0.01, and ***p < 0.001. Error bars represent standard deviation (mean ± SD, *n* = 3). Experiments were performed in biological duplicates and repeated three times. Abbreviations: Esu, sponge *Eunapius subterraneus*; Hsa, *Homo sapiens*

Since human BRMS1 is known to regulate cell migration and colony formation [33, 45], we investigated the role of sponge BRMS1 and human BRMS1, as well as the human BRMS1-like paralog, in these processes in MDA-MB-231 breast cancer cells. Cells were transfected with FLAG-tagged EsuBRMS1, HsaBRMS1, HsaBRMS1-like, or with empty vector (pcDNA3) used as a control. MTT assay revealed that overexpression of all three proteins significantly reduced cell proliferation compared with the empty vector control (*p* < 0.01) (Fig. 6A). We then evaluated their effects on cell survival and colony formation. Colony formation assay showed that overexpression of sponge BRMS1 as well as human BRMS1 and BRMS1-like significantly reduced the number of colonies formed compared with cells transfected with empty vector as a control (*p* < 0.05) (Fig. 6B). To investigate the role of these proteins in cell migration, wound healing and Boyden chamber assays were performed. Wound healing assays showed a significant reduction in cell migration and slower wound closure after 24 h for EsuBRMS1 (*p* < 0.001), HsaBRMS1 (*p* < 0.05), and HsaBRMS1-like (*p* < 0.001) relative to control (Fig. 6C). Boyden chamber assays demonstrated a significant reduction in cell migration for EsuBRMS1, HsaBRMS1, and HsaBRMS1-like overexpressed cells compared with cells transfected with empty vector (*p* < 0.001) (Fig. 6D). These results indicate that sponge BRMS1 and human BRMS1 and BRMS1-like paralogs similarly impact cell viability, proliferation, migration, and colony formation when overexpressed in MDA-MB-231 cells.

## Discussion

In this study, we present the first comprehensive phylogenetic analysis of the BRMS1 protein family, which includes BRMS1, BRMS1-like, and SDS3 proteins [27, 29, 30]. Our results support a duplication of an ancestral *brms1* gene that gave rise to BRMS1 and BRMS1-like, most likely during early vertebrate evolution. Although SDS3 is evolutionarily related to BRMS1 and BRMS1-like, it forms a distinct paralogous lineage. Collectively, these findings indicate that BRMS1 and SDS3 represent ancient paralogs derived from a more distant common ancestor, followed by early lineage-specific divergence within Eumycota and Metazoa. Consistent with the inferred vertebrate-specific duplication of BRMS1, early-branching metazoans such as Porifera harbor only a single BRMS1 homolog. Analysis of domain architecture across Porifera further revealed lineage-specific variation in the presence of the CTD domain. While demosponges, including *E. subterraneus*, lack the CTD domain, it is retained in other sponge classes such as Calcarea and Homoscleromorpha. This pattern suggests either a secondary loss of the CTD domain in the demosponge lineage or an independent acquisition in other sponge lineages. Such lineage-specific differences are in line with previous studies demonstrating extensive gene repertoire variation among sponge classes, and even among demosponges, reflecting the deep evolutionary divergence and genomic plasticity characteristic of Porifera [3, 4, 48, 49].

BRMS1 is a member of the Sin3A/HDAC complex, which is responsible for regulating transcriptional activity in eukaryotic cells [33]. In this complex, human BRMS1 has been shown to be closely interacting with SDS3 (SUDS3) and ARID4A [22, 33], but also to be in close contact with HDAC1 and SIN3A proteins [31]. While the interaction with SIN3A/HDAC has been documented, little is known about the capability of BRMS1 to interact with itself or with BRMS1-like paralog. A potential antiparallel dimerization of BRMS1 is supported by in silico prediction (Additional file 1: Fig. S4), together with a scarce 3D structure of the N-terminal coiled-coil domain of the protein [47]. Furthermore, an analogy can be drawn with the quaternary organization of SDS3 and DEP1 in budding yeast *S. cerevisiae*, two proteins that share a lot of structural similarity with BRMS1, which were found to be interacting in head-to-tail fashion in the resolved 3D structures of yeast SIN3/HDAC complexes (PDB 8i03 and 8hpo) [23, 50].

The inability to purify soluble recombinant EsuBRMS1 or HsaBRMS1 proteins is most likely related to their intrinsically disordered nature, as predicted by AlphaFold3 [51]. Such disorder often promotes aggregation and instability during expression in bacteria, consistent with previous observations for human BRMS1 [52]. Despite our extensive optimization of expression parameters, including temperature, IPTG concentration, growth medium, and bacterial strain, the proteins remained largely insoluble. The best, though still limited, yields were obtained using *Escherichia coli* BL21 CodonPlus (DE3) induced at 30 °C for 3 h with 0.8 mM IPTG. Substantial protein loss during concentration steps further indicated instability, likely reflecting the highly disordered tertiary structure predicted for BRMS1. Similar difficulties were reported previously, as BRMS1 tends to aggregate into inclusion bodies, and even refolding protocols yield minimal amounts of functional protein. To date, BRMS1 has been successfully isolated only as part of the mSin3A–HDAC complex through co-immunoprecipitation [22], while purification of the individual recombinant protein remains a challenge.

Structural and in silico analyses strengthen the possibility that BRMS1 forms antiparallel dimers via conserved coiled-coil domains. The co-immunoprecipitation data obtained in this study are consistent with this model. When expressed in human cells, both EsuBRMS1 and HsaBRMS1-like co-precipitated with HsaBRMS1, indicating physical association among these proteins. This result further confirms that HsaBRMS1 and HsaBRMS1-like are part of the same protein assemblies, likely corresponding to the SIN3/HDAC complex [24].

Remarkably, EsuBRMS1 also interacted with these complexes, suggesting that the sponge homolog is sufficiently similar to human BRMS1 to be recognized and incorporated into the human protein complex. This finding implies a high degree of structural and functional conservation of these proteins. The ability of EsuBRMS1 to integrate into a human chromatin-modifying complex raises the possibility that it might partially compensate for BRMS1 function, at least at the level of physical interaction. Although further analyses are needed to clarify the exact stoichiometry of these interactions, our results provide the first experimental evidence that the oligomerization and complex-forming capacity of BRMS1 is an evolutionarily conserved feature retained from early metazoans, such as sponges.

The BRMS1 protein is primarily localized in the cell nucleus, as it contains nuclear localization signal domains (NLS1 and NLS2) and is part of the Sin3-HDAC chromatin remodeling complex. In addition, it has also been found in the cytoplasm, where it may have additional functions related to cytoskeletal regulation and cell signaling. It is assumed that the intracellular localization of the BRMS1 protein between the nucleus and the cytoplasm influences its ability to suppress metastasis; however, this remains insufficiently studied [35, 53, 54]. Our intracellular localization results confirmed that the sponge homolog, as well as human BRMS1 and BRMS1-like proteins, is localized mainly in the nucleus and partially in the cytoplasm of human MCF-7 and HeLa tumor cells, suggesting evolutionary conservation of their biological function. Although standardized protocols for sponge cell transfection or commercially available sponge cell lines are not yet established, previous studies have demonstrated that heterologous proteins can be expressed in primmorphs, tissue slices, buds, or gemmules of various sponge species, albeit with low efficiency [55–58]. More recently, we successfully transfected primary cell cultures of *E. subterraneus* and determined the intracellular localization of several oncoproteins (MYC, RRAS2, and DRG1), despite the low transfection efficiency (< 1%) [59]. In this study, we determined the intracellular localization of sponge and human BRMS1, apparently both in the nucleus and cytoplasm of transfected sponge cells. Sponge cells do not adhere to the substrate even after treatment with adhesion-promoting compounds (such as poly-L-lysine, laminin, or fibronectin). The resulting cell movement during confocal microscopy makes it difficult to precisely determine the intracellular localization of sponge and human BRMS1 proteins. Despite these technical limitations, our results provide additional evidence for the evolutionary conservation of intracellular localization of BRMS1 proteins in sponge cells and human cancer cells. Since no optimized protocol for sponge cell transfection currently exists, these findings represent an important step toward establishing functional model systems for studying cell biology and molecular mechanisms in sponges.

Our study shows that both sponge BRMS1 and its human orthologs (BRMS1 and BRMS1-like) significantly suppress proliferation, migration, and colony formation in MDA-MB-231 human breast cancer cells. Although human BRMS1 is reported to affect migration and colony formation without significantly altering primary tumor growth [33, 45], our results indicate a reduction of approximately ~ 30% in cell proliferation following overexpression of sponge or human BRMS1 and human BRMS1-like. This effect may reflect a more pronounced protein role during metastatic colonization, when cancer cells proliferate at distant sites after extravasation [60]. We also confirmed that human BRMS1-like reduces migration and colony formation, consistent with previous studies demonstrating its inhibition of EMT and metastasis in breast cancer, and growth suppression in other cancer cell lines [28, 61]. The overlapping intracellular localization and functional effects of BRMS1 and BRMS1-like suggest potential functional redundancy or compensation in human cancer cells. Overall, BRMS1 homologs from sponge and human share conserved functions in tumor-related processes, highlighting the evolutionary conservation of BRMS1 biological activity across metazoans.

## Conclusions

This work highlights four main points: (i) evolutionary analysis indicates that BRMS1 and BRMS1-like arose from a duplication of an ancestral BRMS1 protein, likely during early vertebrate evolution; (ii) sponge BRMS1 and human BRMS1 and BRMS1-like have identical localization, mainly in the nucleus and partially in the cytoplasm, suggesting conservation of their biological function; (iii) sponge BRMS1 protein can coexist in complexes with human BRMS1, indicating an evolutionary conservation sufficient to physically mimic the BRMS1 in human cells, and potentially emulate some of its biological roles normally associated with complex metazoans; (iv) sponge BRMS1 protein, like its human orthologs (BRMS1 and BRMS1-like), significantly suppresses proliferation, migration, and colony formation in MDA-MB-231 human breast cancer cells.

This study demonstrates structural and functional similarities between the BRMS1 protein from the sponge *E. subterraneus* and its human homologs, including conserved intracellular localization and involvement in processes associated with cancer development and progression. The results suggest that the fundamental cellular functions of these proteins were present at the origin of Metazoa and remain conserved between sponges and humans.

## Methods

### Bioinformatics and structural analysis

Protein sequences of BRMS1, BRMS1-like, and SDS3 were acquired from the NCBI database (https://blast.ncbi.nlm.nih.gov/Blast.cgi) using *blastp*. Our evolutionary analysis included protein sequences from selected Metazoa, Filasterea, and Eumycota with publicly available genomes, accession numbers provided in Additional file 2. Sequences containing large insertions or deletions, likely due to misannotation, were excluded from our analysis.

Multiple sequence alignments were performed with ClustalX 2.0 [62] using default parameters. Alignments were edited and shaded in GeneDoc v2.7 [63]. Conserved domains were identified using InterPro [64], NCBI Conserved Domain Database [65], SMART [66], and relevant literature [33, 35]. Phylogenetic relationships were inferred using MEGA7 [67] with a maximum likelihood method calculated using the JTT + G model [68], according to results obtained by ProtTest [69]. Tree robustness was assessed with 1000 bootstrap replicates. Internal branches with bootstrap values greater than 50% were considered reliable subgroups, while lower values were not shown. Amino acid identity and similarity matrices were generated from the multiple sequence alignments using MatGAT 2.01 with BLOSUM62 scoring [70] and visualized as heat maps in Morpheus (https://software.broadinstitute.org/morpheus/).

For intron mapping, annotated nucleotide sequences of *brms1* genes from selected metazoans were acquired from the NCBI genomic database (https://www.ncbi.nlm.nih.gov/genome/). The precise position and phase of each intron were subsequently confirmed through manual verification.

Structure visualization and analysis were performed using Alphafold predicted structures available on the Alphafold database (HsaBRMS1 AF-Q9HCU9-F1; HsaBRMS1-like AF-Q5PSV4-F1; HsaSDS3 AF-Q9H7L9-F1). EsuBRMS1, absent from databases, was internally predicted using Alphafold3. Structures were superimposed and analyzed using Pymol software. Protein sequence and structure alignment was generated using JALVIEW software (*Jalview, ClustalO alignment default settings, identity cutoff 25%; Jalview, secondary structure prediction, JPRED4*). Similarities between HsaBRMS1, HsaBRMS1L, and EsuBRMS1 predicted structures were computed using US-align online software after uploading PDB predicted structures, and using default settings (https://aideepmed.com/US-align/; version 20,241,108; accessed 03/02/2025) [71, 72].

### Plasmid construction

Specific primers were designed to amplify cDNAs corresponding to sponge BRMS1 (EsuBRMS1) and human BRMS1 and BRMS1-like (HsaBRMS1 and HsaBRMS1-like) proteins. EsuBRMS1 (accession number YAA50088.1) was amplified from the cDNA library of *E. subterraneus*, while the human homologs were amplified from commercially available plasmids (RC203428 and RC202645, Origene). EsuBRMS1, HsaBRMS1, and HsaBRMS1-like were amplified, sequenced, and cloned into various vectors (pEGFPN1, pmCherryC1, and pcDNA3.1) using specific primers and restriction enzymes listed in Additional file 1: Table S2. The resulting constructs were GFP-, CHERRY-, or FLAG-tagged, depending on the specific experiment.

### Cell culture and transfection procedure

MCF-7 (ECACC cat. no. 86012803) and MDA-MB-231 (ATCC cat. no. HTB-26) cell lines were maintained in Dulbecco’s Modified Eagle Medium with high glucose (DMEM, Sigma-Aldrich) supplemented with 10% fetal bovine serum (FBS, Capricorn Scientific), 1% nonessential amino acids (Sigma-Aldrich), and 1% antibiotic/antimycotic solution (Capricorn Scientific) at 37 °C in a humified atmosphere containing 5% CO_2_. For transfection, cells were seeded into 6-, 24-, or 96-well plates in a growth medium and allowed to adhere for 24 h. Transfections were performed using Lipofectamine 2000 or Lipofectamine 3000 (Thermo Fisher Scientific) according to the manufacturer’s instructions, and cells were subsequently incubated for 24 or 48 h.

The Ogulin cave sponge *Eunapius subterraneus* was collected from the Tounjčica cave near Ogulin, Croatia. This species was selected due to the easy availability during the favorable hydrological conditions in the caves and the availability of genomic data from the Bioinformatics Group, University of Zagreb. *E. subterraneus* is closely related to *Ephydatia muelleri* [73], an emerging sponge model [4]. Sponges were maintained in cave water and incubated at 8 °C with occasional aeration. Primary sponge cell cultures were prepared, and cells were transfected with the plasmid of interest using Turbofect (Thermo Fisher Scientific), as previously described (Dominko et al., 2023).

### Confocal microscopy

Confocal microscopy was performed on MCF-7, MDA-MB-231 cells, and sponge cells. Cells were transfected with EsuBRMS1, HsaBRMS1, and HsaBRMS1-like fluorescently labeled with GFP or CHERRY. In addition, co-transfections were carried out for all pairwise combinations of these three proteins. The experiments were carried out following previously established protocols [20, 21, 59]. In short, the cells grown on coverslips were washed three times in the PBS and fixed with 4% sucrose/paraformaldehyde for 15 min. Sponge cells were not fixed. Cell nuclei were depicted with Hoechst stain. Confocal images were acquired using the laser scanning confocal microscope Leica TCS SP8 (Leica Microsystems, Wetzlar, Germany). Further image processing was conducted using the ImageJ software (National Institutes of Health).

### SDS-PAGE and western blot analysis

Extracted proteins from cultured cell lines were quantified using the Pierce BCA protein assay kit (Thermofisher, 23,227) according to the manufacturer’s instructions. Even amounts of proteins were loaded onto freshly prepared 12% polyacrylamide gels (Bio-Rad), separated by SDS-PAGE and transferred to PVDF membranes (Roche). To confirm equal loading of proteins, the membranes were stained with AmidoBlack (Sigma-Aldrich) [20, 21]. Upon blocking in 0.2% (w/v) I-block (Thermo Fisher Scientific), the membranes were incubated with the appropriate primary antibodies, followed by secondary antibodies (anti-flag (Mo; Sigma-Aldrich, #F1804); anti-brms1 (Rb; Abcam, ab134968); anti-mouse-HRP (Cell Signaling Technology, #7076S); anti-rabbit-HRP (Invitrogen, #31,460); anti-GFP (Rb; ChromoTek, pabg1)). Chemiluminescence signals were visualized using an ECL blotting substrate (GE Healthcare) and captured on the Alliance Q9 mini imaging platform (UVITEC Cambridge). All raw images of western blot data are shown in Additional file 4.

### Co‑immunoprecipitation

For detection of proteins interacting with BRMS1, 5 × 10^5^ of MCF-7, MDA-MB-231, or HEK293 cells were seeded in a six-well plate and (co)-transfected to express GFP (∅), HsaBRMS1-GFP (H1G), HsaBRMS1-like-GFP (H1LG), EsuBRMS1-GFP (E1G), HsaBRMS1-FLAG (H1F), and EsuBRMS1-FLAG (E1F) in different combinations. Cells were transfected using Lipofectamine 3000 (Thermo Fisher Scientific) according to the manufacturer’s instructions, collected 24 h after transfection, lysed in co-IP buffer (50 mM Tris pH 7.4, 150 mM NaCl, 2 mM EDTA, 1% NP40, 0.5% Triton X-100) supplemented with protease inhibitor cocktail (Roche Applied Science), and sonicated with two bursts of 10 s on ice. Total protein concentration was measured using the commercially available Pierce BCA protein assay kit (Thermo Fisher Scientific) according to the manufacturer’s protocol. Immunoprecipitation of FLAG-tagged EsuBRMS1 or HsaBRMS1 was performed using a magnetic Flag Agarose (Pierce A36797) according to the manufacturer’s instructions. In all steps, beads were collected using a Dynamag magnet (Thermofisher). Briefly, 10 µL of slurry was washed 3 times in 1 mL TBS (50 mM Tris HCl pH7.4, 150 mM NaCl). About 250 µg of extracted protein was incubated with the washed beads in a final volume of 200 µL (completed with TBS), overnight on a rotator at + 4 °C. Beads were washed 3 times for 15 min on a rotator at room temperature with 1 mL of TBST (TBS supplemented with 0.1% Tween20). Proteins were eluted by resuspending the beads in 20 µL of 2X Loading buffer (2XLB) followed by a 10-min incubation at 95 °C. Beads were separated and the supernatant was immediately loaded on SDS-PAGE for western blot analysis.

### MTT assay

For cell proliferation analysis using the MTT assay (3-(4,5-dimethylthiazol-2—yl)−2,5-diphenyltetrazolium bromide; Millipore), 4 × 10^3^ MDA-MB-231 cells per well were seeded in a 96-well plate. Cells were then transfected with EsuBRMS1-FLAG, HsaBRMS1-FLAG, or HsaBRMS1-like-FLAG constructs as previously described [20, 21]. Forty-eight hours after transfection, the growth medium was removed and 1 × MTT solution was added. Following a 4—h incubation, DMSO was added and gently mixed for 2 min. Absorbance was measured at 570 nm using an ELISA microplate reader (LabSystem Multiskan MS, Artisan Technology Group). Absorbance values, reflecting the metabolic activity of viable and proliferating cells, were normalized to the empty vector control.

### Colony formation assay

For the colony formation assay, MDA-MB-231 cells were initially seeded into a 6-well plate and transfected with EsuBRMS1-FLAG, HsaBRMS1-FLAG, or HsaBRMS1-like-FLAG constructs, and further processed following a previously established protocol [21]. Twenty-four hours after transfection, cells were seeded into 60—mm dishes at a density of 5 × 10^4^ cells/dish. G418 (Neomycin, Sigma-Aldrich) was added to a final concentration of 500 µg/mL. After 10 days, colonies were fixed, air-dried, stained with Giemsa (Sigma-Aldrich), and counted. Colonies were defined as Giemsa-stained cell clusters visible to the naked eye after 10 days of G418 selection. Colonies were manually counted, and the total number per dish was normalized to the empty vector control.

### Cell migration assays

For monitoring the migratory capacity of human cell lines, wound healing and Boyden chamber assay were used. A wound healing assay was performed by seeding 5 × 10^4^ MDA-MB-231 cells into a 24-well plate, transfected with an EsuBRMS1-FLAG, HsaBRMS1-FLAG, or HsaBRMS1-like-FLAG construct and further processed as already described [21]. Twenty-four hours post-transfection, a linear scratch was introduced into the cell monolayer. Cells were rinsed with fresh medium and incubated for an additional 24 h. Cell migration was evaluated by measuring the distance between the wound edges at T0 and T24 hours in five randomly selected fields per chamber using 100 × magnification (Olympus CKX41, Tokyo, Japan). The cell-free area was quantified by comparing the initial area at 0 h with the area remaining after 24 h at the same location within the well, using ImageJ software (National Institutes of Health, USA). For the Boyden chamber assay, MDA-MB-231 cells were seeded into a 6-well plate, transfected with the aforementioned constructs and processed as already described [21]. Shortly, 24 h after transfection, cells were seeded into Transwell migration inserts (8 µm pore size; Corning) in the serum free medium at a density of 2.5 × 10^4^ cells/well and allowed to migrate toward 10% FBS in DMEM. Migrated cells on the underside of the membrane were fixed with 4% paraformaldehyde and stained with 1% crystal violet. Images were captured at 200 × magnification using a microscope (Olympus BX51, Tokyo, Japan), and cell numbers were quantified using ImageJ software (National Institutes of Health, USA). Results were normalized on the control (empty vector).

### Statistical analysis

Statistical analyses were performed using SPSS for Windows (v17.0). All biological experiments were conducted in two or three biological replicates and repeated at least three times to ensure the reliability of the results. Western blot and other biological assay results were quantified using ImageJ software (NIH). Differences between two independent groups were assessed using Student’s *t*-test, while comparisons among multiple groups were analyzed by one-way ANOVA. Statistical significance was set at *p* < 0.05 for all analyses.

## Supplementary Information

Additional file 1: Fig. S1 Phylogenetic analysis of BRMS1, BRMS1-like, and SDS3 proteins from selected Metazoa and Eumycota. Fig. S2 Heatmap of amino acid sequence similarity and identity for BRMS1 and BRMS1-like proteins from selected species. Fig. S3 Structural representation of conserved amino acid between HsaBRMS1 and EsuBRMS1. Fig. S4 Alphafold3 prediction of oligomerization structures of HsaBRMS1. Fig. S5 Flag-immunoprecipitation of human and sponge BRMS1-FLAG in A) MDA-MD-231 and B) HEK293 cell lines. Fig. S6 The subcellular localization of the proteins EsuBRMS1 and HsaBRMS1 in human HeLa tumor cells. Fig. S7 Positive and negative control of sponge cells transfection. Table S1 Structural similarities between pairs of BRMS1 proteins. Table S2 Primers used for cloning the brms1 cDNAs into expression vectors.

Additional file 2: Accession numbers and amino acid sequences of BRMS1, BRMS1-like, and SDS3 proteins from selected species.

Additional file 3: The amino acid sequence identity and similarity percentages of BRMS1 homologs from selected species.

Additional file 4: Original data of western blots.

## Data Availability

All the data generated or analyzed during this study are included in this published article and its supplementary information files.

## Abbreviations

BRMS1: Breast cancer metastasis suppressor 1
NME1: NME/NM23 nucleoside diphosphate kinase 1
FAU: FAU ubiquitin like and ribosomal protein S30
DRG1: Developmentally regulated GTP binding protein 1
RRAS2: RAS related 2
Sin3-HDAC complex: The switch-independent 3 (SIN3)/histone deacetylase (HDAC) complex
SDS3: SIN3A corepressor complex component SDS3
NLS: Nuclear localization signals
NES: Nuclear export signal
EGFR: Epidermal growth factor receptor
NF-κB: Nuclear factor kappa B
FAK: Focal adhesion kinase
uPA: Plasminogen activator, urokinase
CXCR4: C-X-C motif chemokine receptor 4
OPN: Osteopontin
PI3K: Phosphoinositide 3-kinase
Akt: Serine/threonine kinase
Src: SRC proto-oncogene, non-receptor tyrosine kinase
CTD: C-terminal capped Tudor domain
GFP: Green fluorescent protein
CHERRY: Red fluorescent protein
Hsa: *Homo sapiens*
Esu: *Eunapius subterraneus*
