# Supplementary material for: A sponge homolog of BRMS1 reveals ancient origin of metastasis-suppressing functions
Source: BMC Biol. 2026 May 5;24:118. doi: 10.1186/s12915-026-02616-5 (PMC13173789; doi:10.1186/s12915-026-02616-5)
Supplement: Supplementary file 1 — Additional file 1: Fig. S1 Phylogenetic analysis of BRMS1, BRMS1-like, and SDS3 proteins from selected Metazoa and Eumycota. Fig. S2 Heatmap of amino acid sequence similarity and identity for BRMS1 and BRMS1-like proteins from selected species. Fig. S3 Structural representation of conserved amino acid between HsaBRMS1 and EsuBRMS1. Fig. S4 Alphafold3 prediction of oligomerization structures of HsaBRMS1. Fig. S5 Flag-immunoprecipitation of human and sponge BRMS1-FLAG in A) MDA-MD-231 and B) HEK293 cell lines. Fig. S6 The subcellular localization of the proteins EsuBRMS1 and HsaBRMS1 in human HeLa tumor cells. Fig. S7 Positive and negative control of sponge cells transfection. Table S1 Structural similarities between pairs of BRMS1 proteins. Table S2 Primers used for cloning the brms1 cDNAs into expression vectors. [file 12915_2026_2616_MOESM1_ESM.pdf]

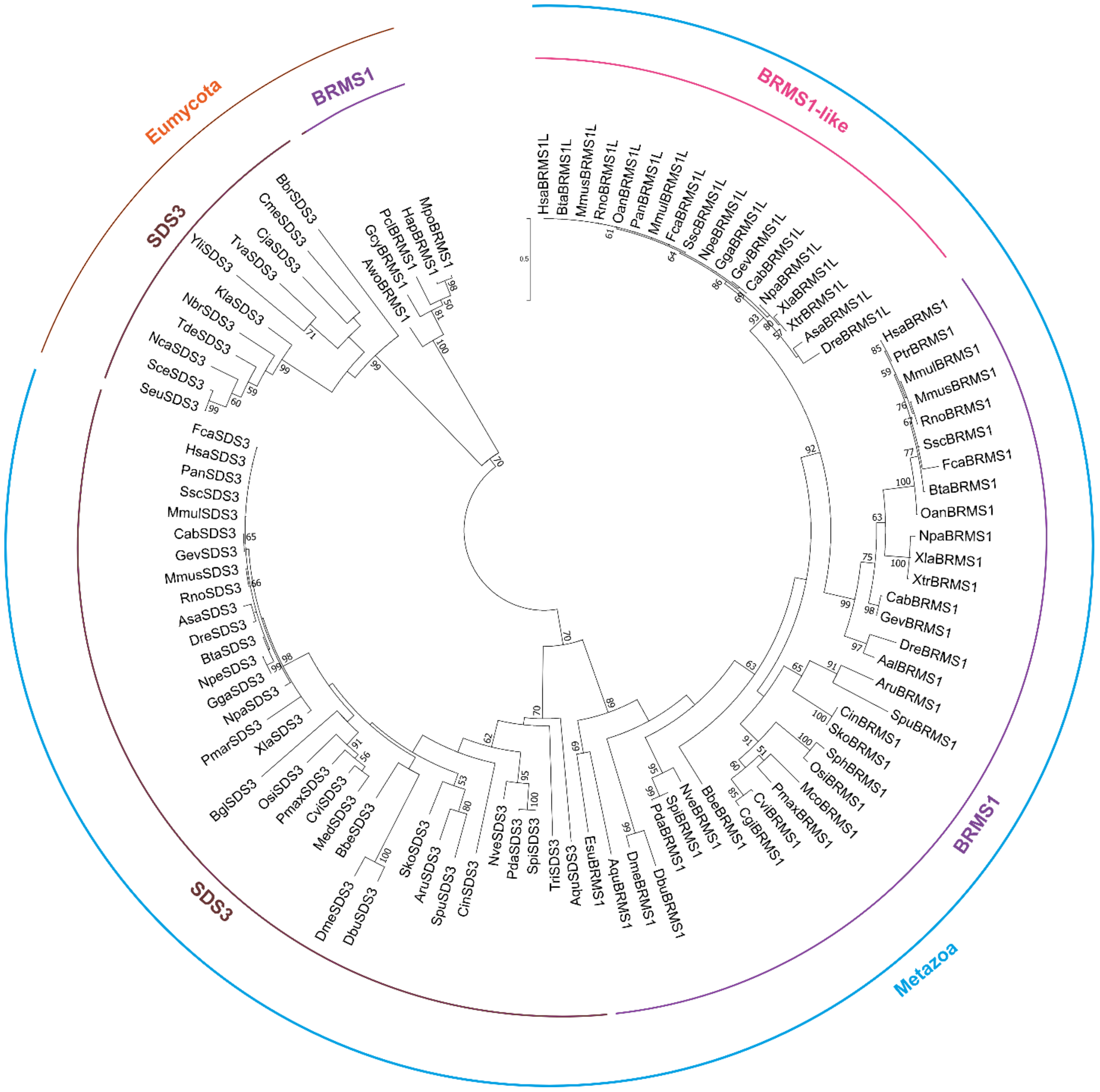

Figure S1. Phylogenetic analysis of BRMS1, BRMS1-like, and SDS3 proteins from selected Metazoa and Eumycota. The phylogenetic tree was constructed using the maximum likelihood method (JTT+G model). Bootstrap support values, based on 1000 replicates, are shown at the branching points (only values >50% are displayed). The scale bar indicates the number of substitutions per site. Representatives from Eumycota are shown in orange and from Metazoa in blue. SDS3

proteins are shown in brown, BRMS1 proteins in purple and BRMS1-like proteins in pink. Abbreviations and accession numbers are listed in Additional file 2.

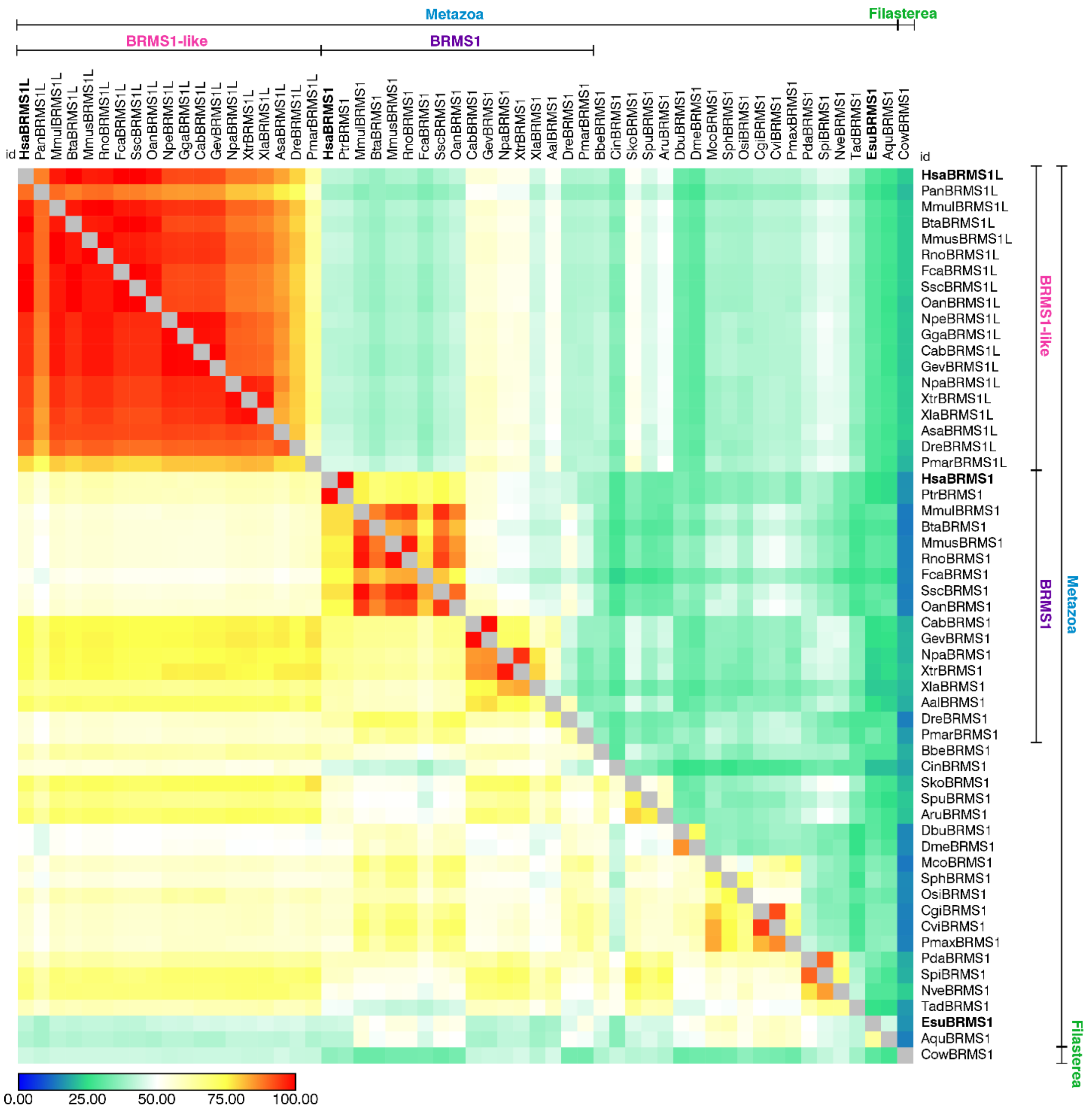

Fig. S2. Heatmap of amino acid sequence similarity and identity for BRMS1 and BRMS1-like proteins from selected species. A heatmap is presented to visualize the protein sequence similarity (lower left) and identity (upper right) values. Warm colors (yellow and red) indicate high amino acid similarity (> 50%), while cool colors (green and blue) represent low similarity (< 50%). Abbreviations and accession numbers are listed in Additional file 2. The corresponding

identity/similarity percentage matrices (calculated using MatGAT2.01 with Matrix BLOSUM62 scores) can be found in Additional file 3.

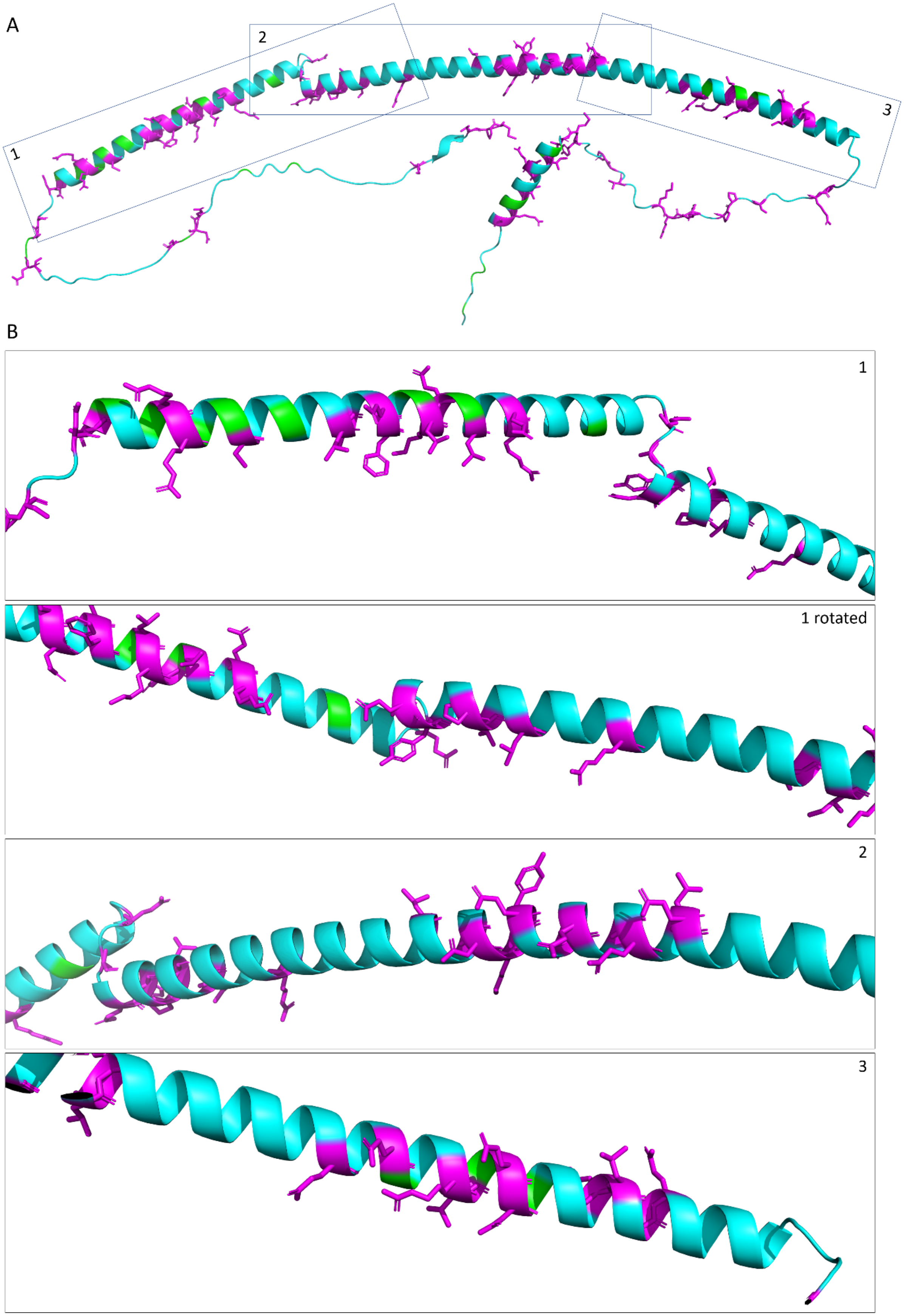
A
1
2
3
B
1
1 rotated
2
3

Fig. S3. Structural representation of conserved amino acid between HsaBRMS1 and EsuBRMS1. A) 3D structure of HsaBRMS1 (AF-Q9HCU9) displaying the exact same amino acids (magenta) and similarly charged amino acids (green, i.e. D<>E and R<>K) as conserved with EsuBRMS1. B) Zoom on the different parts of the structure.

It is to note that most of the conserved amino acids are located in alpha helixes. The unstructured N-terminus of the proteins shares little conservation with EsuBRMS1. Along the first alpha helix, the conserved amino acids are in the majority polarized toward one side of the helix, creating a surface of conserved amino acids (B1). On the second alpha helix, three hotspots are found to display conserved amino acids. The first one orients the amino acids on a specific surface similar to the orientation of alpha Helix1 (B1 rotated). The two other hotspots seem to conserve amino acids strictly, cycling around the helix (B2 and B3). Finally, the C-terminus of the protein displays a high amount of conserved amino acids, especially in the third Helix. The conserved amino acids are keeping the same relative position and surface-orientation on the predicted EsuBRMS1 structure.

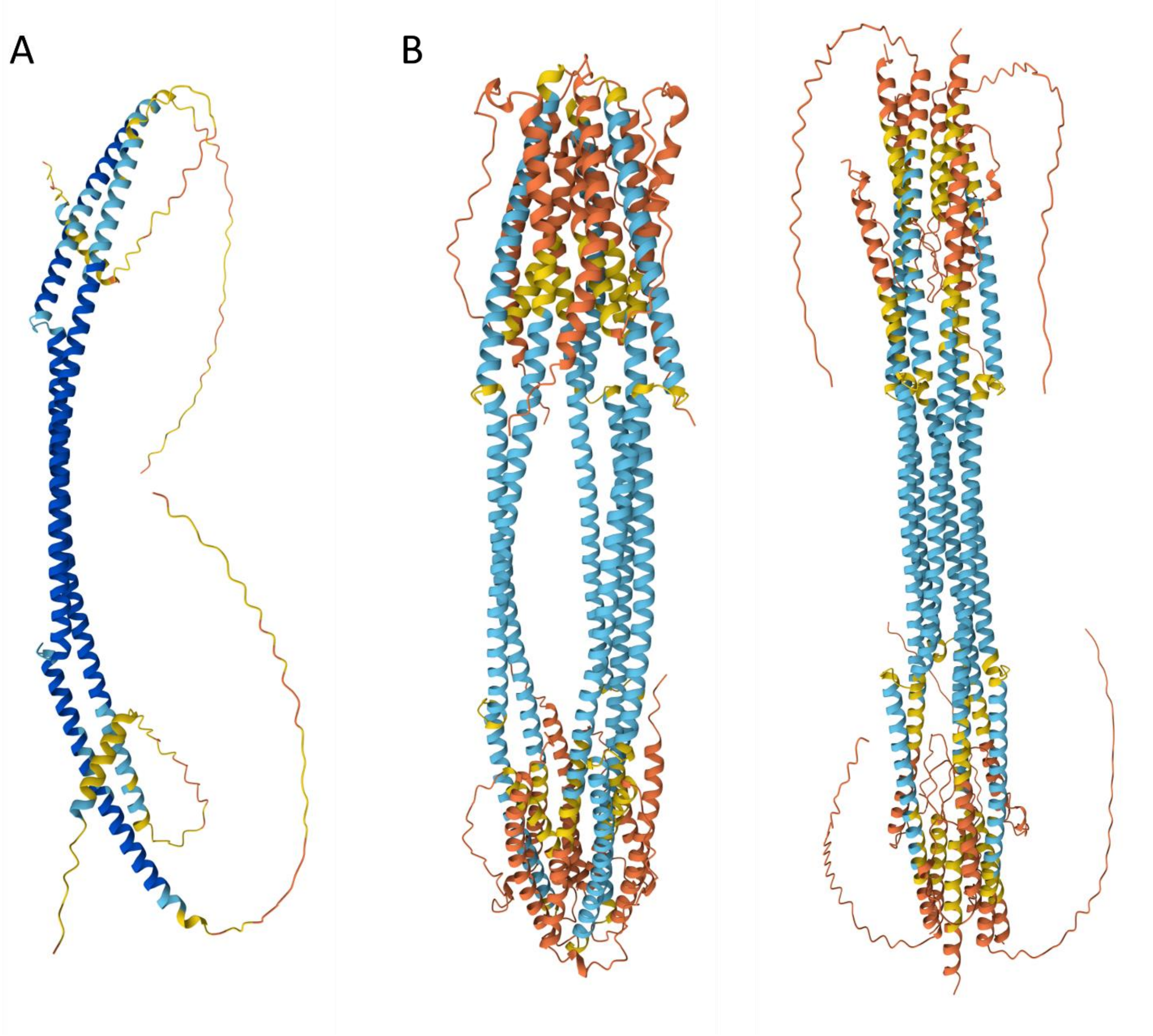

Fig. S4. Alphafold3 prediction of oligomerization structures of HsaBRMS1. Structures are colored by pLDDT confidence (Very High_Dark blue: >90; Confident_Light blue: 90>pLDDT>70; Low_Yellow: 70>pLDDT>50; Very Low_Orange: pLDDT<50). A) Representative 3D structure of dimerization prediction (pTM=0.46, ipTM=0.46). B) Representative 3D structures of hexamerization prediction. (pTM=0.22, ipTM=0.17). A pTM score measures the accuracy of the entire structure, and when above 0.5 the structure folding might be considered as similar to a true

structure. The iPTM score reflects rather the inter-chain interface accuracy within complexes, with a score above 0.8 representing a confident high-quality prediction. In our results, the dimer structures obtained a pTM and ipTM just below 0.5, suggesting that the overall folding might be correct. On the other hand, the hexameric structures displayed a very low scores, highlighting the unlikeliness of this oligomerization to exist. More strikingly, in the dimer structure the proteins interact in a head-to-tail fashion, and the pLDDT score of the first two alpha helixes reflect high confidence (dark blue). The hexameric prediction results in a drop of pLDDT for each monomer, indicating again a lower probability for that structure to exist. Notably, the two main hexameric organizations, trimer-of-dimers or straight hexamers, always involve the interaction of monomers in head-to-tail fashion. Altogether, the predicted structure suggests that the oligomerization of HsaBRMS1 is most likely to happen as a dimer oriented in head-to-tail fashion, rather than as a hexamer.

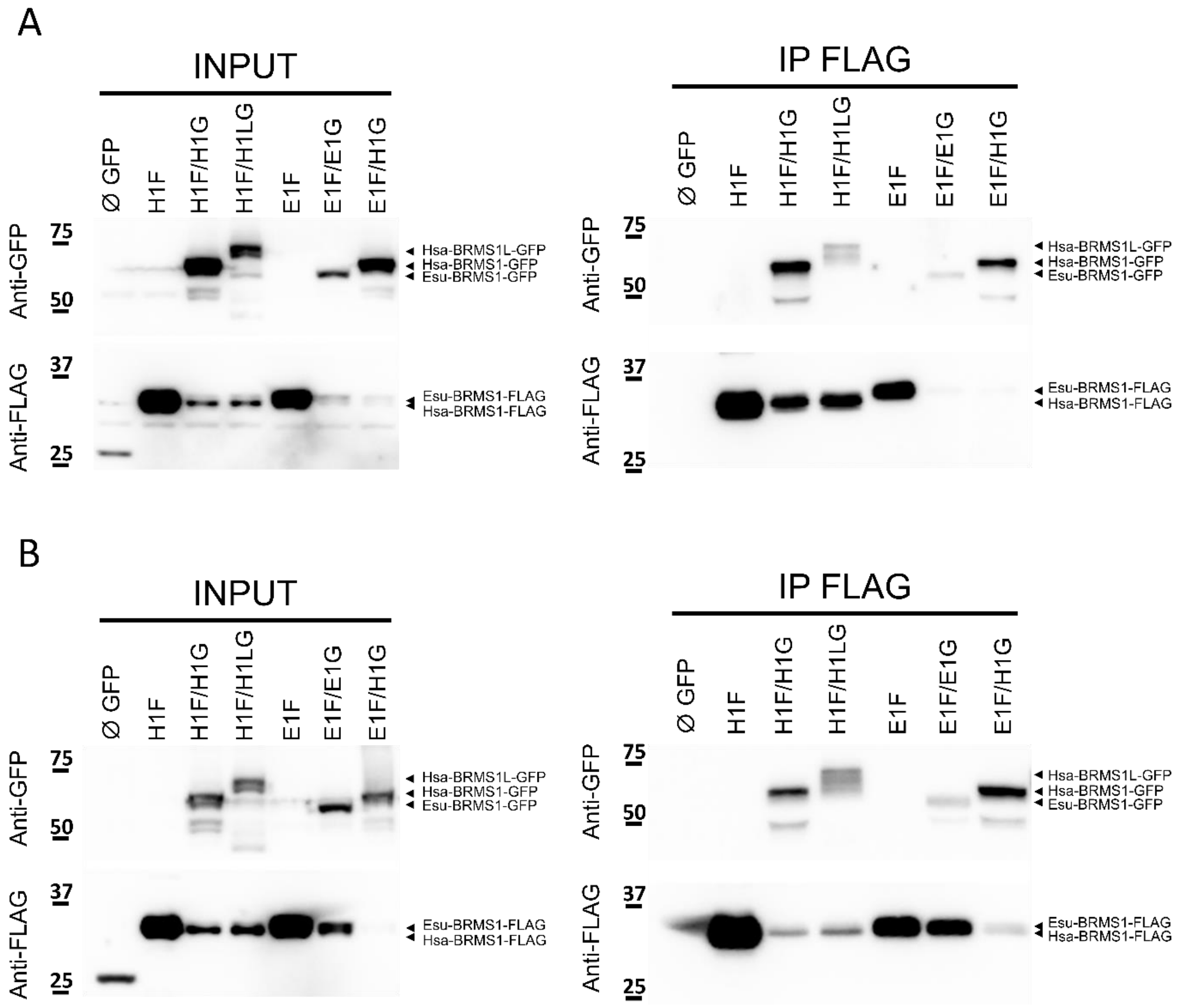

Fig. S5. Flag-immunoprecipitation of human and sponge BRMS1-FLAG in A) MDA-MD-231 and B) HEK293 cell lines. Sponge BRMS1 interacts with itself and human BRMS1. Similarly, human BRMS1 interacts with both itself and BRMS1-like. MDA-MD-231 and HEK293 cells were (co)transfected with plasmids encoding empty GFP (Ø GFP), human proteins HsaBRMS1-FLAG (H1F), HsaBRMS1-GFP (H1G), HsaBRMS1-like-GFP (H1LG) or sponge proteins EsuBRMS1-FLAG (E1F) and EsuBRMS1-GFP (E1G). Co-immunoprecipitated proteins were analyzed by western blot using anti-FLAG and anti-GFP antibodies. Abbreviations: Esu – sponge *Eunapius subterraneus*; Hsa – *Homo sapiens*.

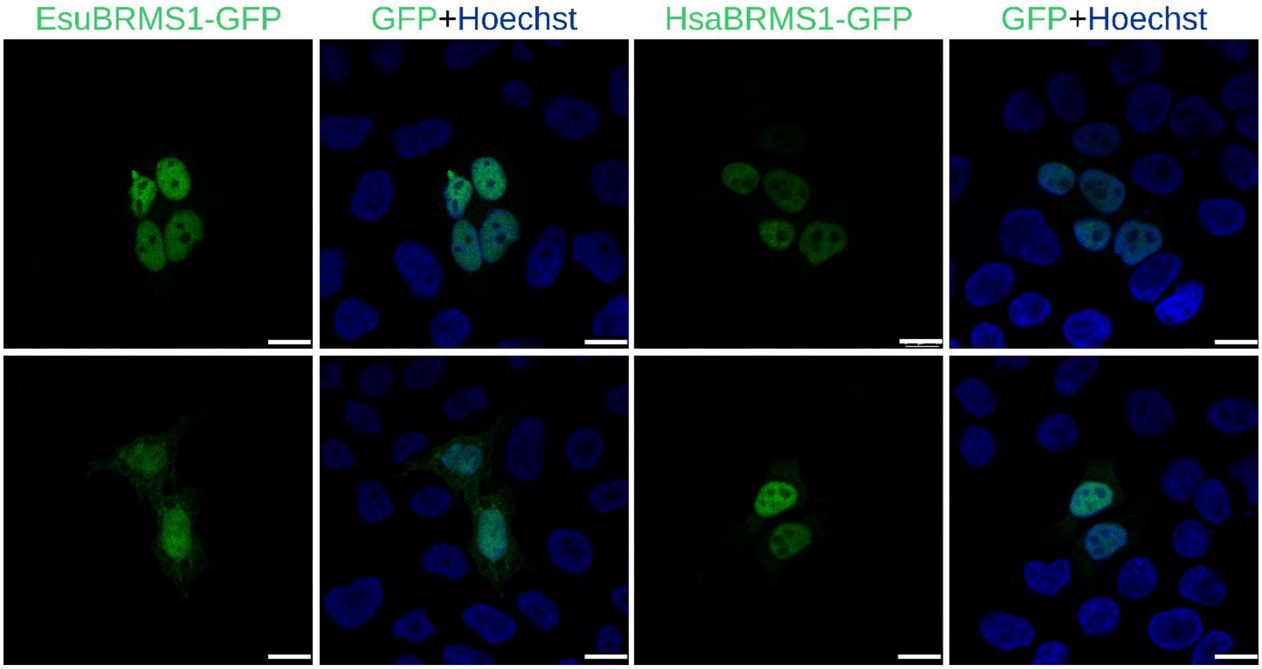

Fig. S6. The subcellular localization of the proteins EsuBRMS1 and HsaBRMS1 in human HeLa tumor cells. The subcellular localization is predominantly nuclear, but is also present in the cytoplasm. EsuBRMS1 and HsaBRMS1 were labeled with GFP (green). Nuclei were counterstained with Hoechst (blue). Cells were analyzed using confocal microscopy. Transfection efficiency >50%. Scale bar represents 10 µm. Abbreviations: Esu – sponge *Eunapius subterraneus*; Hsa – *Homo sapiens*.

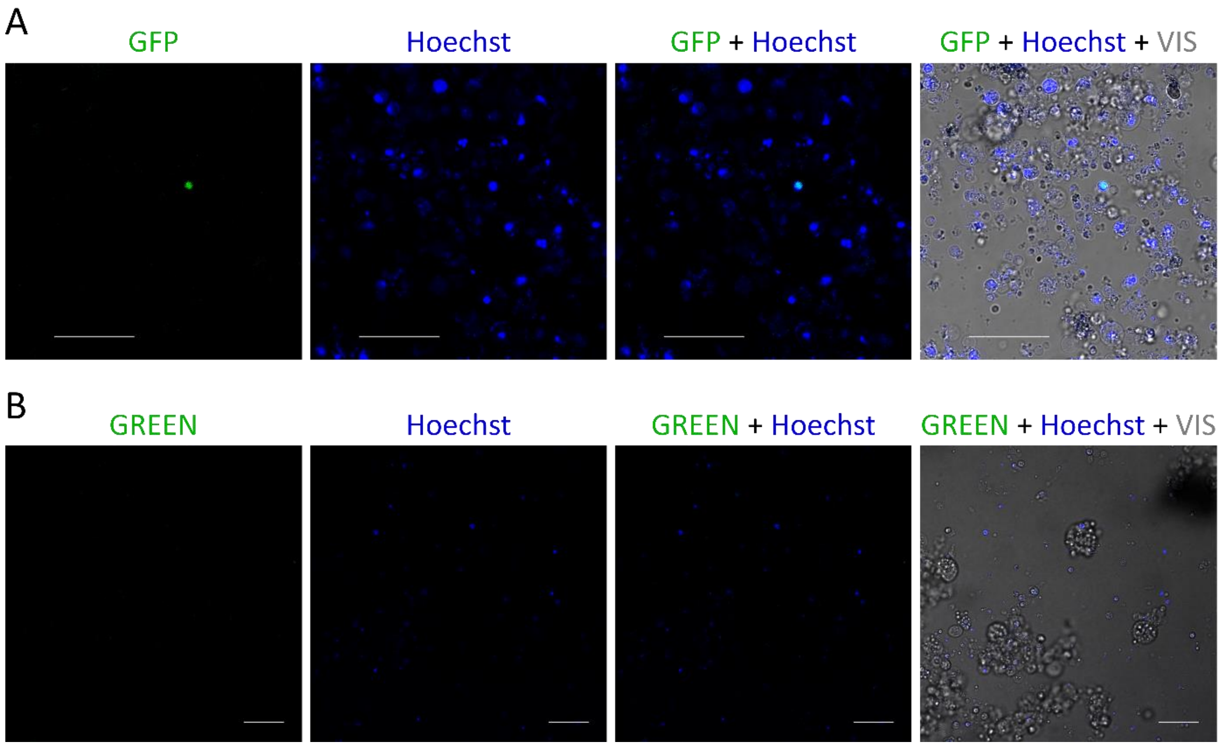

Fig S7. Positive and negative control of sponge cells transfection. A) Sponge cells transfected with an empty pEGFP plasmid to express raw GFP protein. The transfection efficiency is below 1%. B) Sponge cells transfected only with the transfection reagent without plasmid acted as negative control. The autofluorescence in the green channel is negligible. Nuclei were counterstained with Hoechst (blue). Scale bar represents 25 µm.

Table S1. Structural similarities between pairs of BRMS1 proteins

**HsaBRMS1 vs EsuBRMS1**						
	Length (aa)	Aligned length (aa)	SeqID	D0	TMscore	RMSD
Normalized on HsaBRMS1 length	246	187	0.273	5.81	0.57026	4.38
Normalized on EsuBRMS1 length	230	187	0.273	5.63	0.60334	4.38
**HsaBRMS1L vs EsuBRMS1**						
	Length (aa)	Aligned length (aa)	SeqID	D0	TMscore	RMSD
Normalized on HsaBRMS1L length	323	164	0.366	6.57	0.43952	3.24
Normalized on EsuBRMS1 length	230	164	0.366	5.63	0.59746	3.24

<u>Structural similarities between pairs of BRMS1 proteins, computed using USalign online software</u> (https://aideepmed.com/US-align/; Version 20241108; Accessed 03/02/2025). The Alphafold predicted PDB structures of HsaBRMS1, HsaBRMS1L and EsuBRMS1 were uploaded and the software was run using default settings. In both cases, the 3D structure comparison results in the proper alignment of the first two alpha helixes structures (187 and 164 aa aligned), with most of the amino acid pairs being in a 5 Angstrom vicinity. Even though the sequence identity remain low (around 27%), when comparing HsaBRMS1 and EsuBRMS1 the RMSD remains under 5Angstrom which is common for homolog proteins, and the TM-Scores are elevated, with a score above 0.5. Altogether, it indicates that the two proteins fall in the same-fold family and support the distant homolog relationship between the two proteins. The comparison between HsaBRMS1L and EsuBRMS1 is harder to interpret, due to the C-terminal extension domain of the human protein. The aligned sequences corresponding to the first alpha helixes displayed a higher sequence identity (around 37%) with a lower RMSD (3.24angstrom), indicating a tight structural conservation of those helixes. However, the TM-scores are drastically fluctuating depending on

the normalization upon the longer or the shorter protein, since the length of the protein directly influences the calculation of the TM. Hence, the normalization upon HsaBRMS1L reduces the TM-score to fall under the 0.5 cutoff, highlighting a structural difference that is essentially the result of the C-terminal extra domain of HsaBRMS1L protein. Altogether, the first two alpha helixes are fairly conserved between HsaBRMS1, HsaBRMS1L and EsuBRMS1, but HsaBRMS1 and EsuBRMS1 are more structurally related because they do not display the extra C-terminal domain that can be found in HsaBRMS1L.

Table S1. Primers used for cloning the brms1 cDNAs into expression vectors

**brms1 cDNA**	**Vector/ tag**	**Primers/ restriction sites**
brms1 (Esu)	/	5'-ATGCCGGTCAACAGTACAACGAAAAG-3'<br>5'-GTTACAGTTCTACCACCTTTCTGTG-3'
brms1 (Esu)	pEGFP-N1 GFP (C-terminus)	EcoRI: 5'-GTCTAGGAATTCATGCCGGTCAACAG-3'<br>BamHI: 5'-CTAGACGGATCCCGGGACAGTTCTACCAC-3'
brms1 (Hsa)	pEGFP-N1 GFP (C-terminus)	XhoI: 5'-GTCTAGCTCGAGATGCCAGTCCATTCCCGAGGGG-3'<br>EcoRI: 5'-CTAGACGAATTCCGGATGAATGTTTAATTGAATATTTTCC-3'
brms1 (Hsa)	pmCherry-C1 CHERRY (N-terminus)	XhoI: 5'-GTCTAGCTCGAGGCATGCCTGTCCAGCCTCCAAGC-3'<br>EcoRI: 5'-CTAGACGAATTCTTAAGGTCCATCCGATTTTCTCTTC-3'
brms1-like (Hsa)	pmCherry-C1 CHERRY (N-terminus)	XhoI: 5'-GTCTAGCTCGAGGCATGCCAGTCCATTCCCGAGG-3'<br>EcoRI: 5'-CTAGACGAATTCTTATGAATGTTTAATTGAATATTTTCC-3'
brms1 (Esu)	pcDNA3.1 flag (N-terminus)	BamHI: 5'-GTCTAGGGATCCATGGACTACAAGGACGACGACGATAAGATGCCGGTCAACAGTACAACG-3'<br>EcoRI: 5'-CTAGACGAATTCTTACAGTTCTACCACCTTTCTG-3'
brms1 (Hsa)	pcDNA3.1 flag (N-terminus)	EcoRI: 5'-GTCTAGGAATTCATGGACTACAAGGACGACGACGATAAGATGCCTGTCCAGCCTCCAAGC-3'<br>XhoI: 5'-CTAGACCTCGAGTTAAGGTCCATCCGATTTTCTC-3'
brms1-like (Hsa)	pcDNA3.1 flag (N-terminus)	KpnI: 5'-GTCTAGGGTACCATGGACTACAAGGACGACGACGATAAGATGCCAGTCCATTCCCGAGGGGATAAGAAGGAGA-3'<br>XhoI: 5'-CTAGACCTCGAGTTATGAATGTTTAATTGAATATTTTCCTTTCTGTAGCTG-3'

Esu – *Eunapius subterraneus*

Hsa – *Homo sapiens*
