# Supplementary figures and images for: A sponge homolog of BRMS1 reveals ancient origin of metastasis-suppressing functions
Source: BMC Biol. 2026 May 5;24:118. doi: 10.1186/s12915-026-02616-5 (PMC13173789; doi:10.1186/s12915-026-02616-5)

INPUT

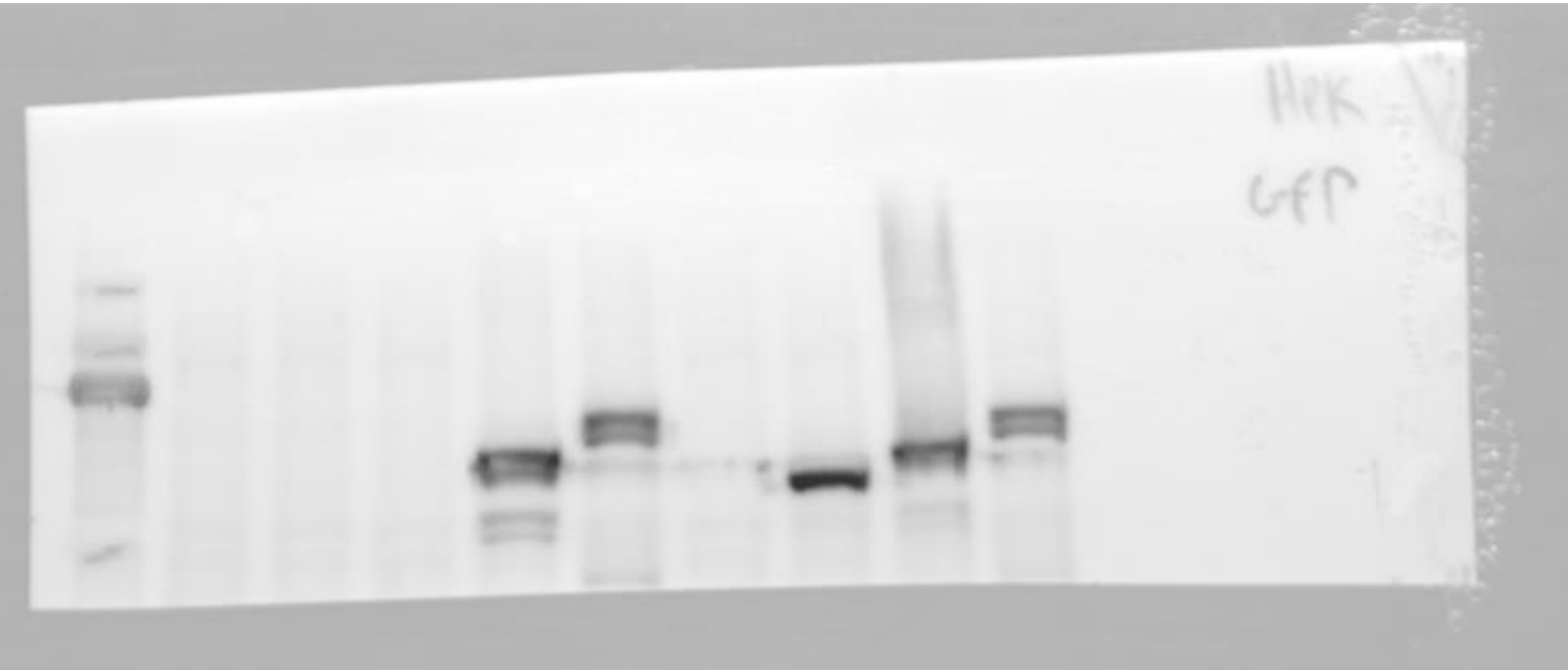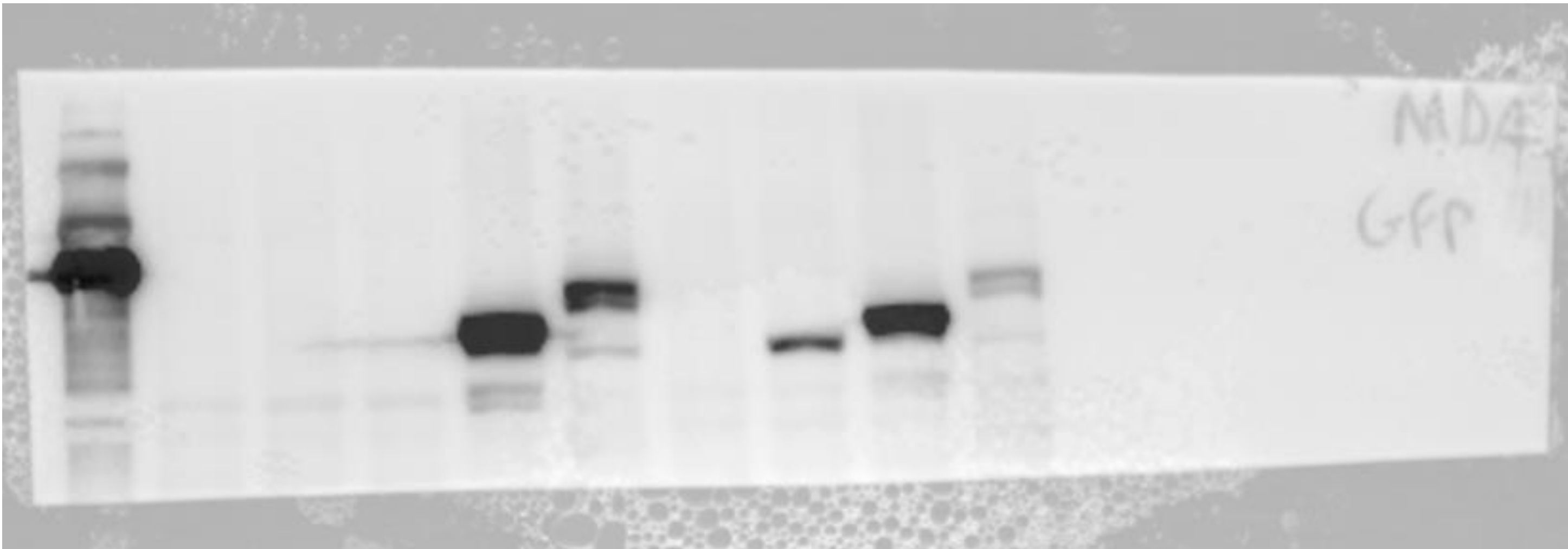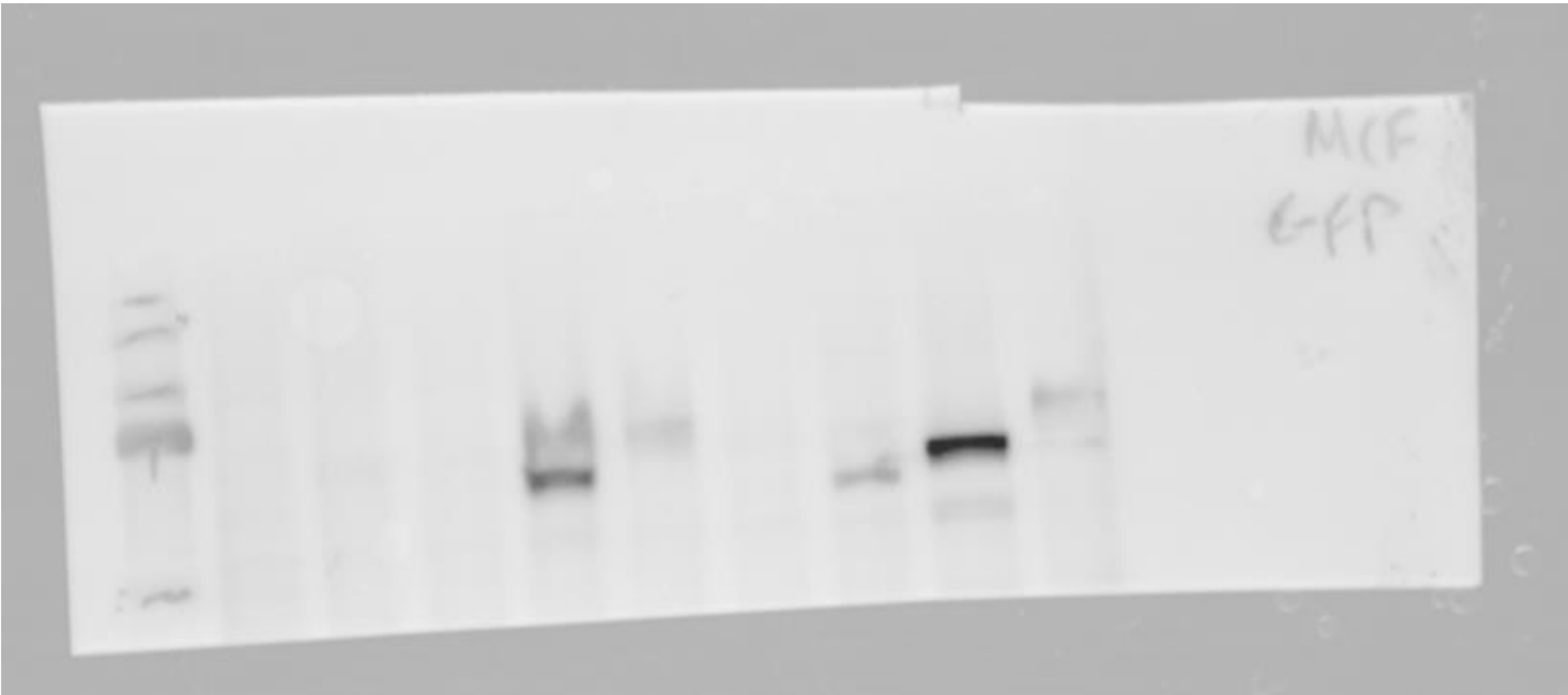

Anti GFP

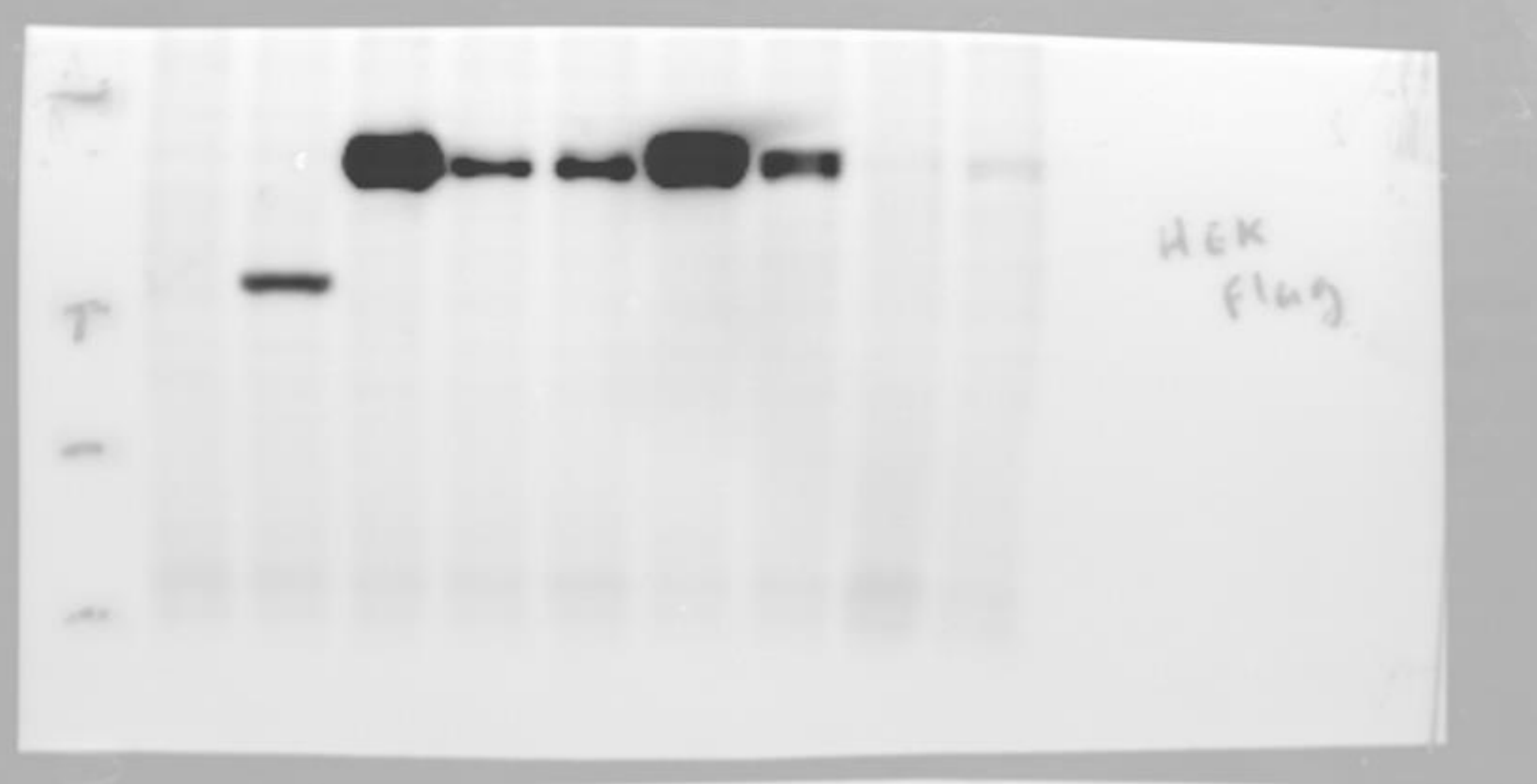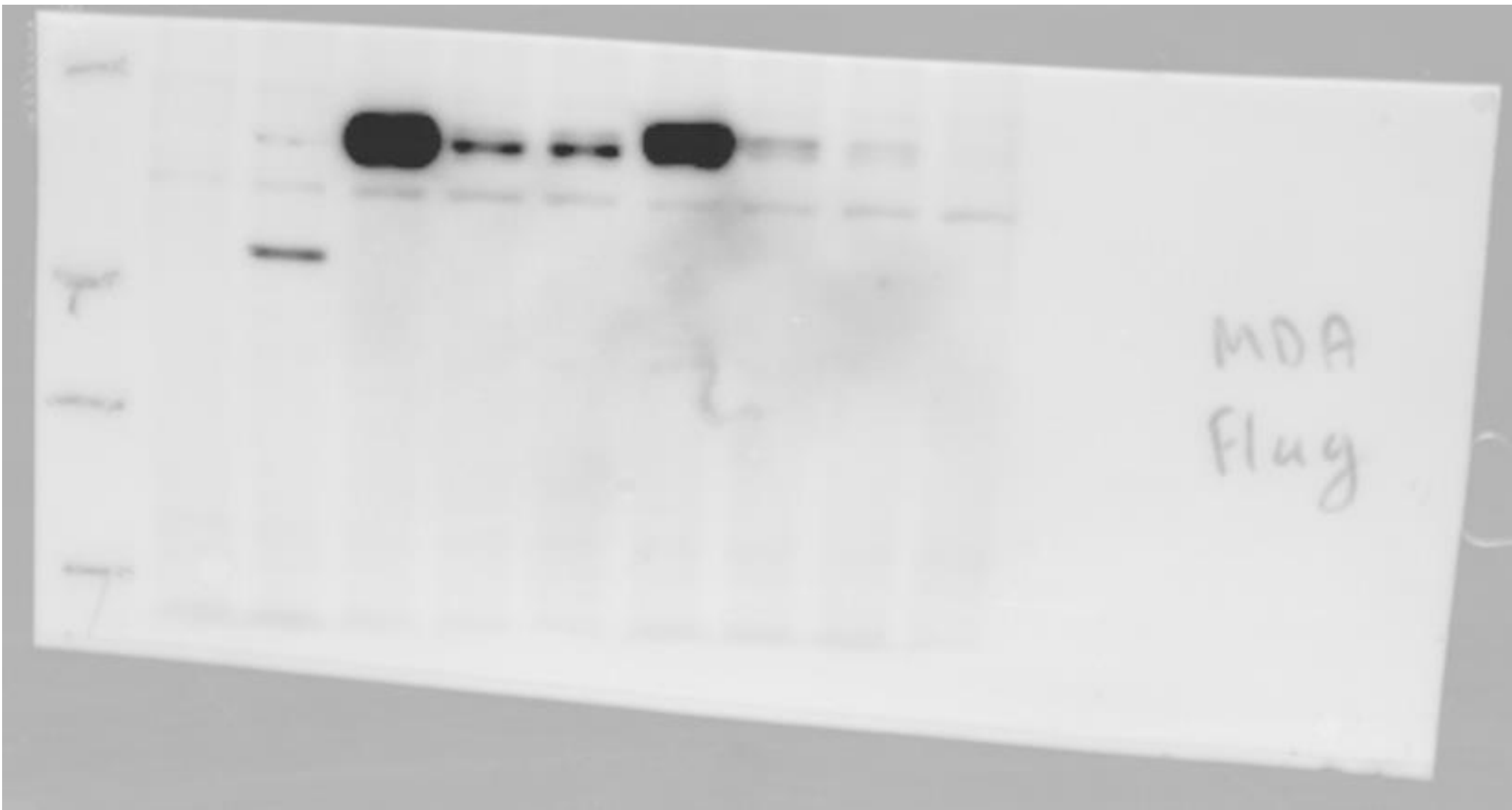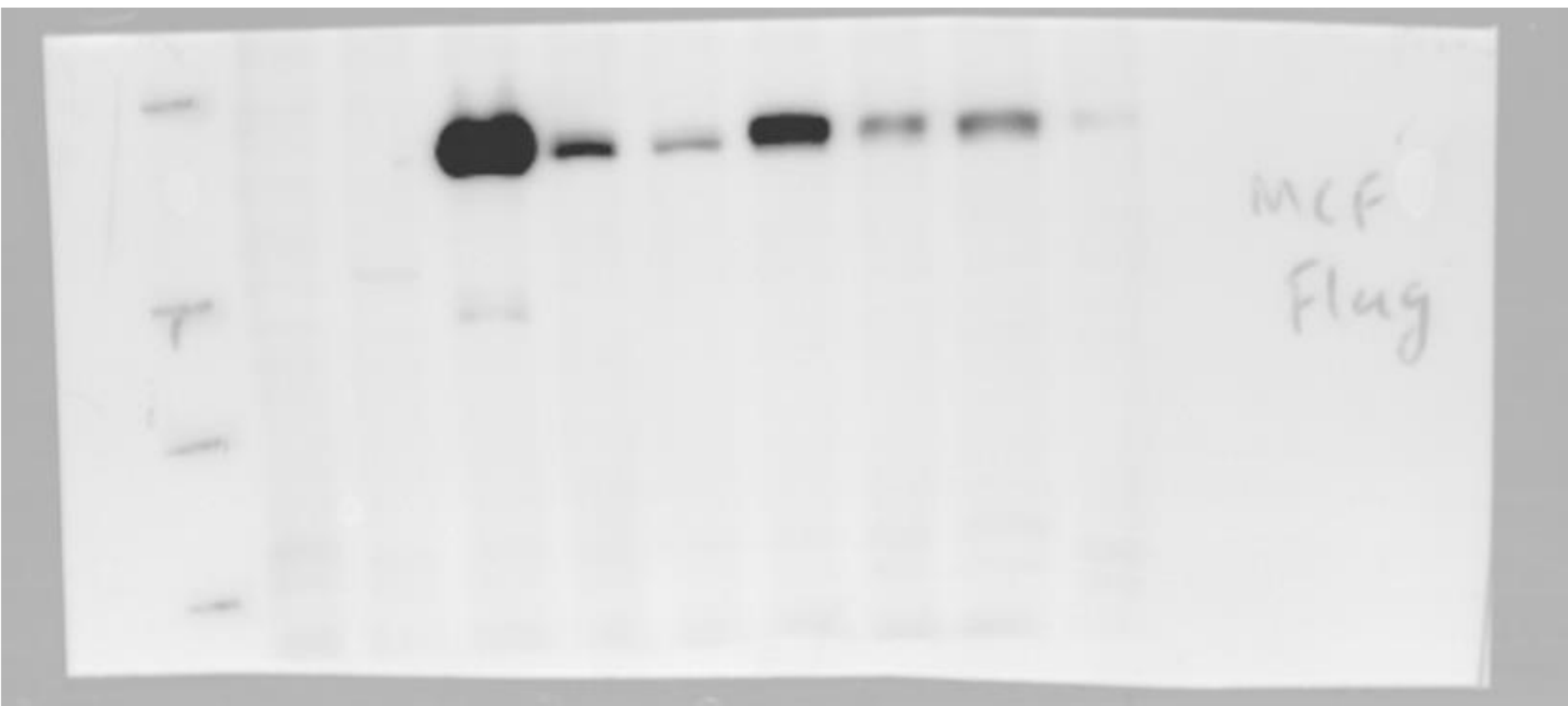

Anti FLAG

HEK293

MDA-MB-231

MCF7

IP

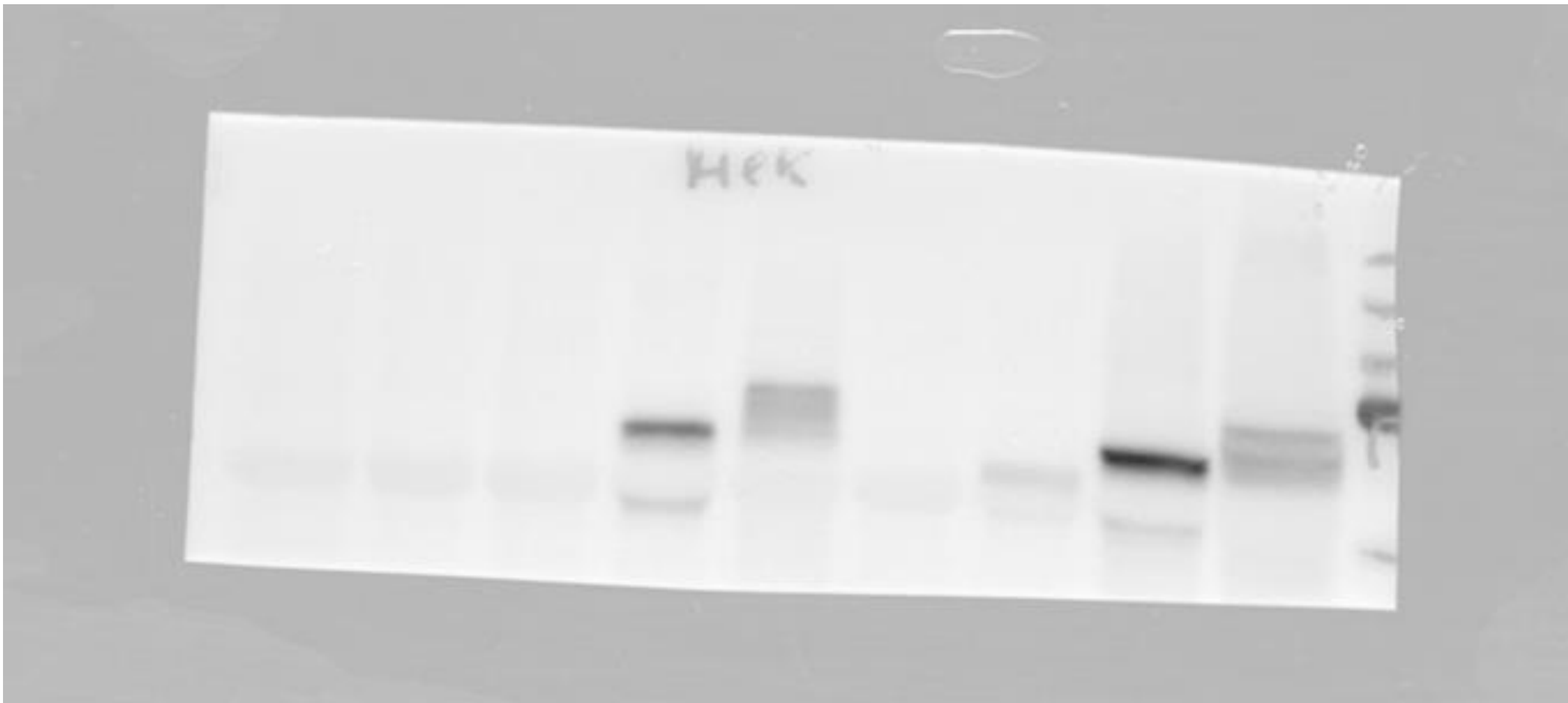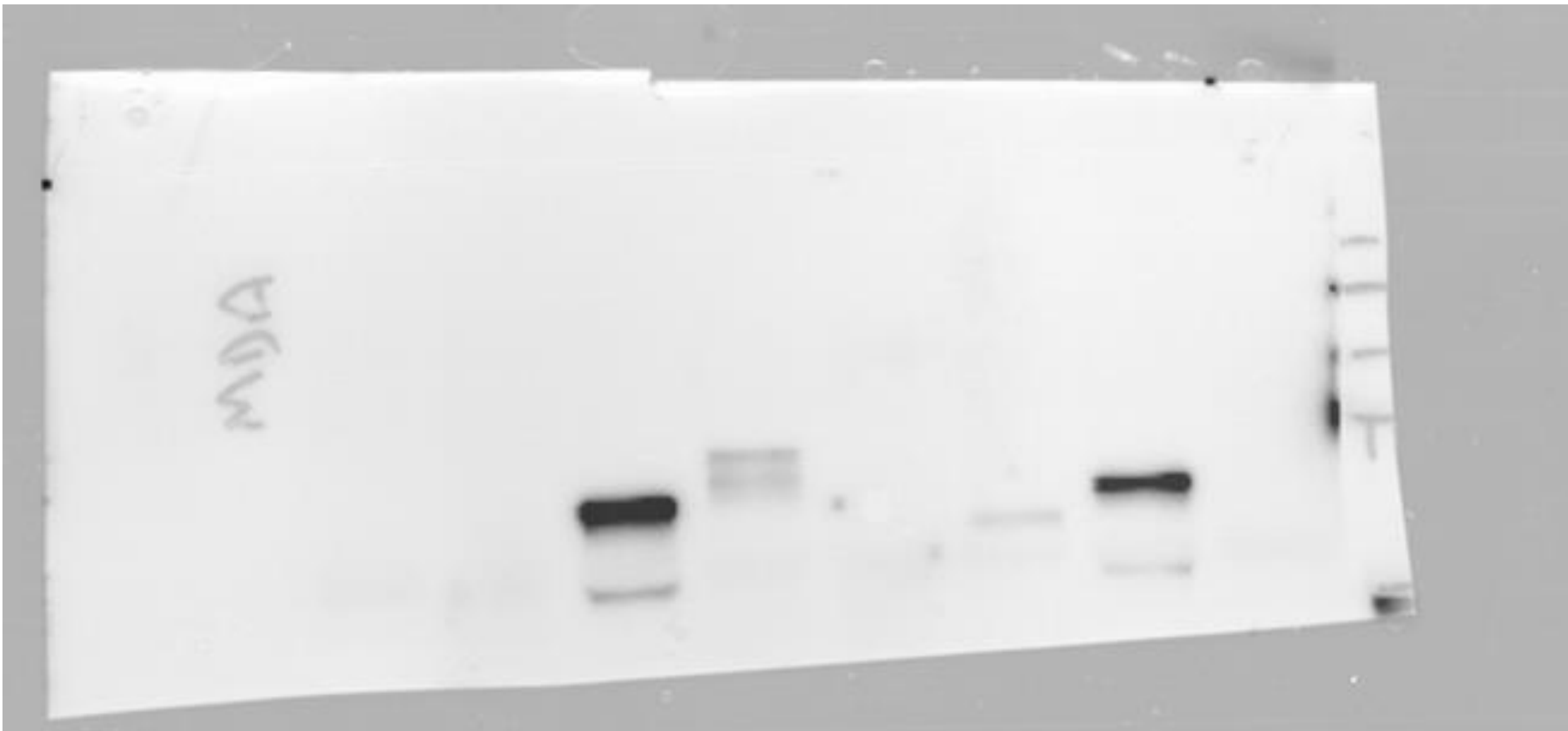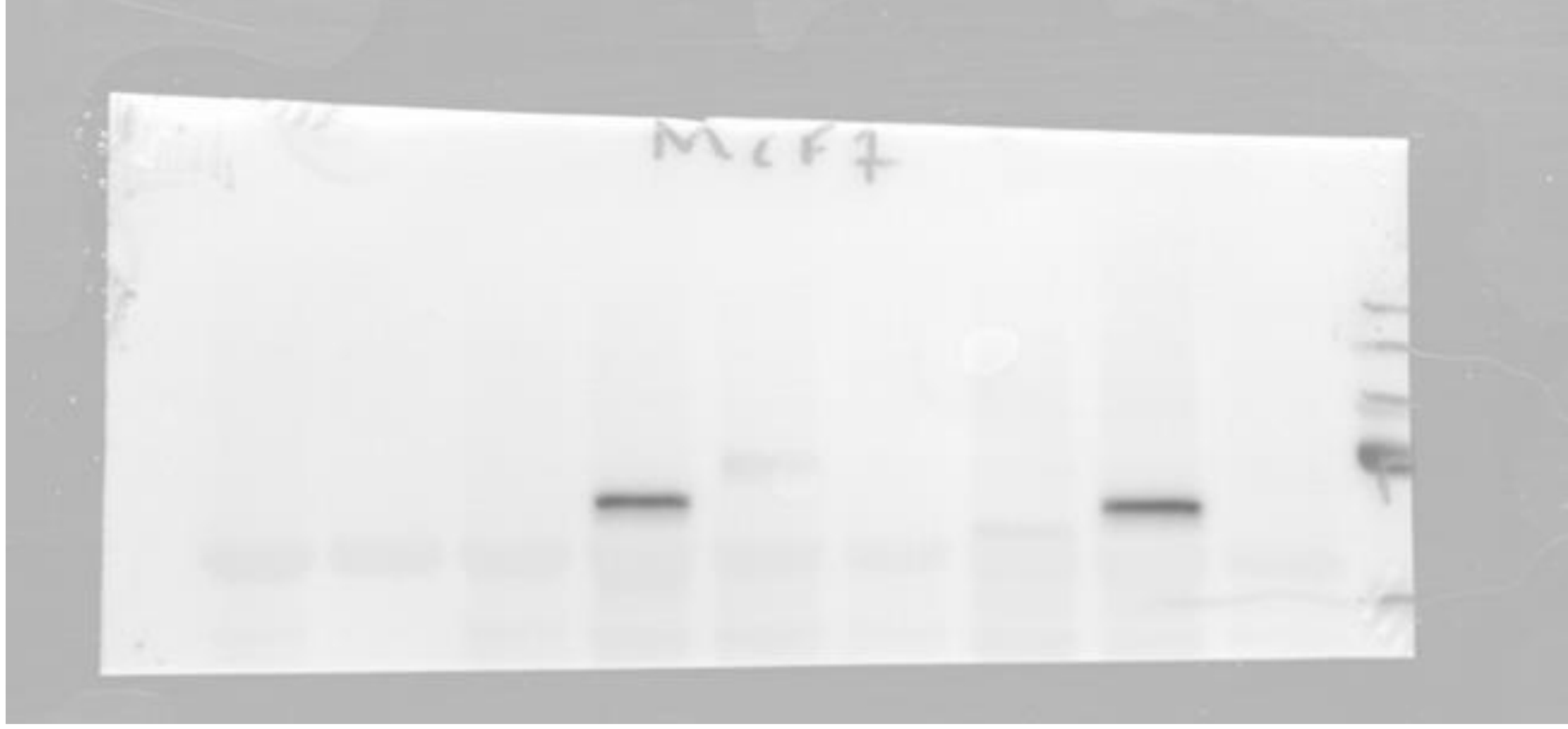

Anti GFP

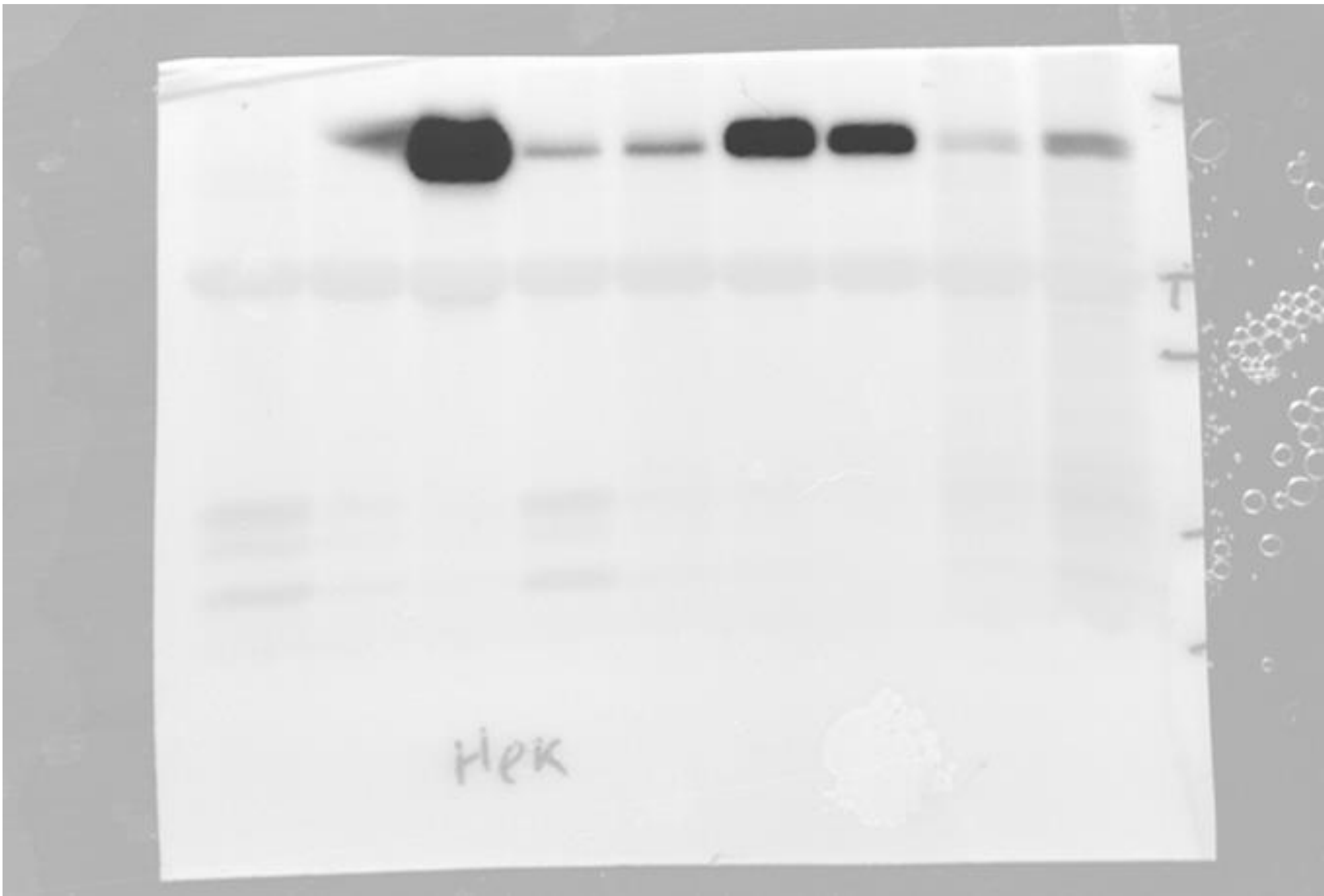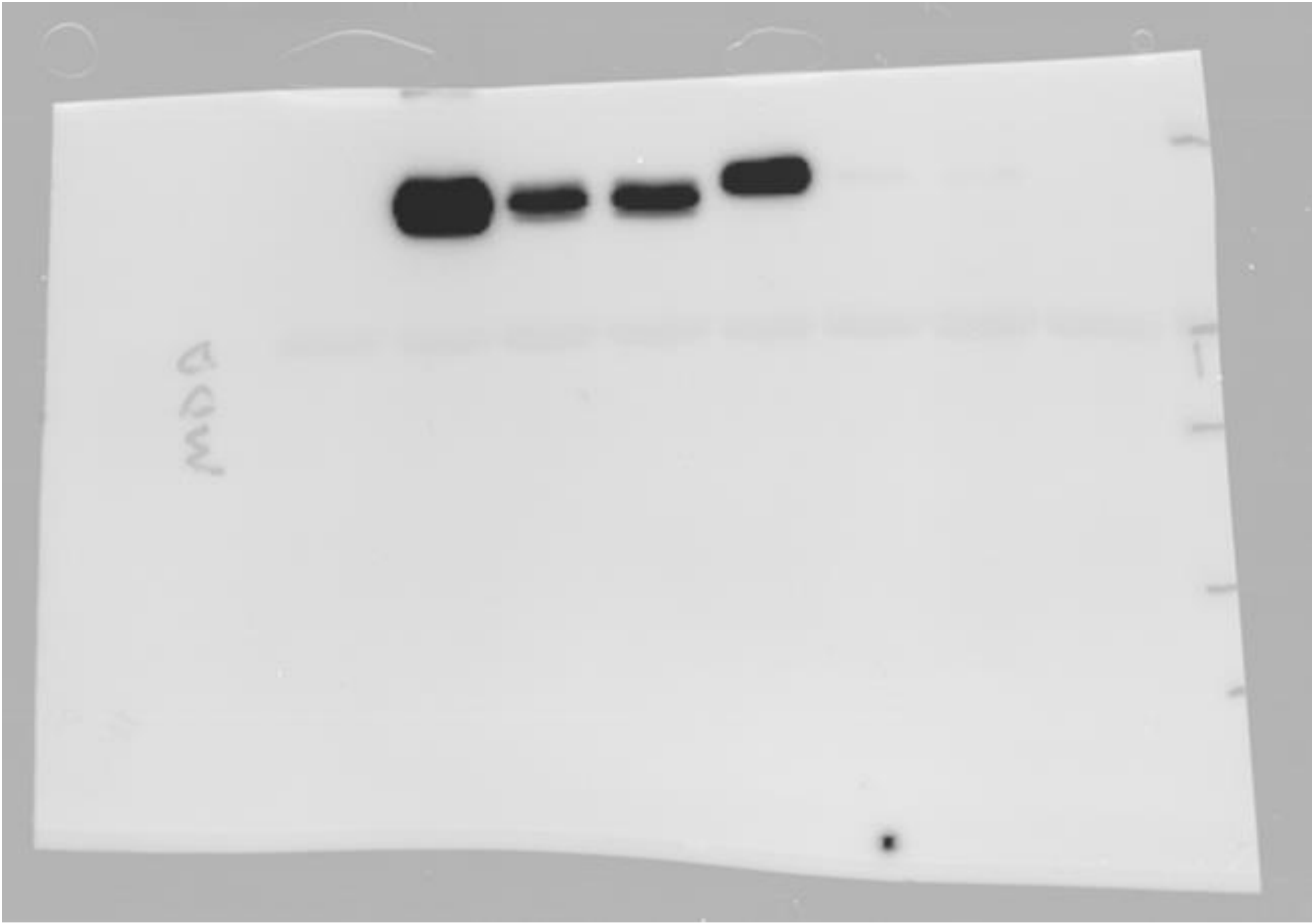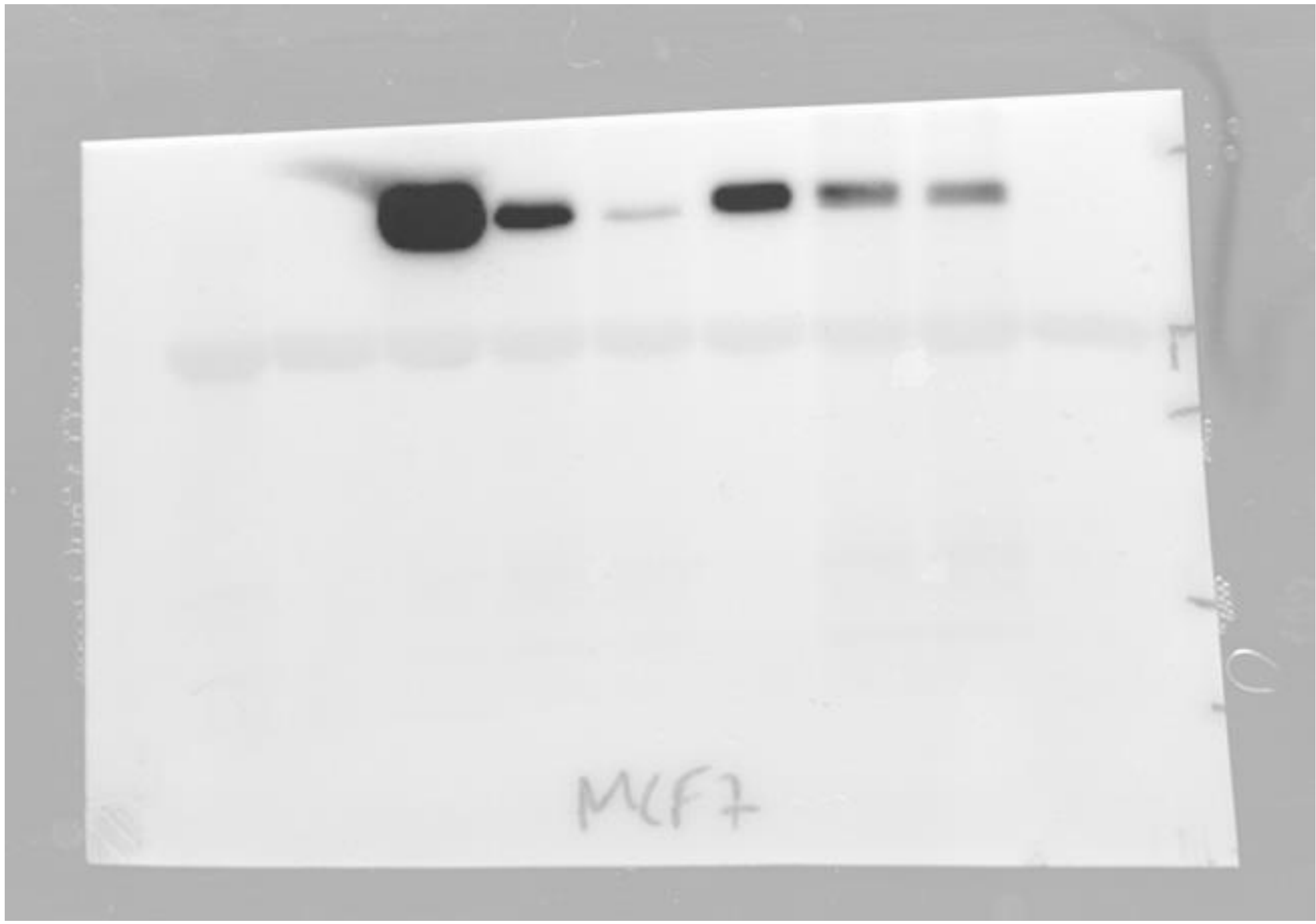

Anti FLAG

HEK293

MDA-MB-231

MCF7

Supplement: Supplementary file 4 — Additional file 4: Original data of western blots. [file 12915_2026_2616_MOESM4_ESM.pdf]
